# Repulsive inverse-distance interatomic interaction from many-body quantum electrodynamics

**DOI:** 10.1126/sciadv.aee5141

**Published:** 2026-08-28

**Authors:** Loris Di Cairano, Matteo Gori, Reza Karimpour, Alexandre Tkatchenko

**Affiliations:** Department of Physics and Materials Science, University of Luxembourg, L-1511 Luxembourg City, Luxembourg.

## Abstract

Interactions between objects can be classified as fundamental or emergent. Fundamental interactions are either extremely short-range or decay inversely with the separation distance, such as the Coulomb potential between charges or the gravitational attraction between masses. In contrast, emergent quantum van der Waals (vdW) and Casimir interactions decay considerably faster (*R*^−6^ or *R*^−7^) with distance *R*. Here, we apply perturbative quantum electrodynamics (QED) to a many-body (MB) system of atoms modeled as charged harmonic oscillators, and reveal a persistent inverse-distance MB-QED interaction stemming from the coupling between virtual photons and molecular plasmons in the nonretarded regime. This interaction, scaling with the third power of the fine-structure constant, is reminiscent of the Lamb shift for a single atom. Although weaker than vdW forces, this MB-QED *R*^−1^ interaction may substantially surpass gravitational attraction in future experiments probing quantum gravity at microscopic scales.

## INTRODUCTION

The quantum vacuum, as the ground state of interacting fields, gives rise to both fundamental forces between charges and emergent forces between neutral systems (*1*–*4*). Quantum electrodynamics (QED), regarded as one of the most successful and precise theories in physics, produced an exquisitely accurate description of the interactions between charged particles and the electromagnetic field (EMF) (*2*, *3*, *5*–*8*). However, only recently has QED been extended to treat realistic molecules and materials composed of many particles (*9*–*11*). This is a very productive and intriguing direction of research that has yielded seminal results, especially for matter in cavities and under a strong coupling regime (*12*–*19*).

Much less is known about the weak-coupling effects of QED for many-body (MB) quantum matter; in particular, can one expect emergent interactions when coupling many interacting atoms or molecules with the EMF? Conventional molecular QED that treats atoms as isolated dipoles perturbatively coupled to the EMF has been very successful. However, a rigorous and unified treatment of electromagnetic interactions between multiple molecules has remained intractable (*5*). In addition, at short distances and minimal coupling, the scalar charge-charge interaction is stronger than the dynamic charge-EMF interaction; thus, perturbative QED must be used with care. In this work, we combine the many-body dispersion (MBD) formalism (*20*, *21*), which captures the collective electronic response in molecular systems to infinite order, with perturbative QED, resulting in a unified theory we refer to as many-body QED (MB-QED). In the MBD framework, atoms are modeled as quantum Drude oscillators (QDOs) coupled via dipolar interactions. MBD is fully general because it provides a transferable and accurate description of collective electronic fluctuations and is applicable in chemistry, biology, nanoscale systems, and condensed matter (*22*, *23*).

**Fig. 1.**
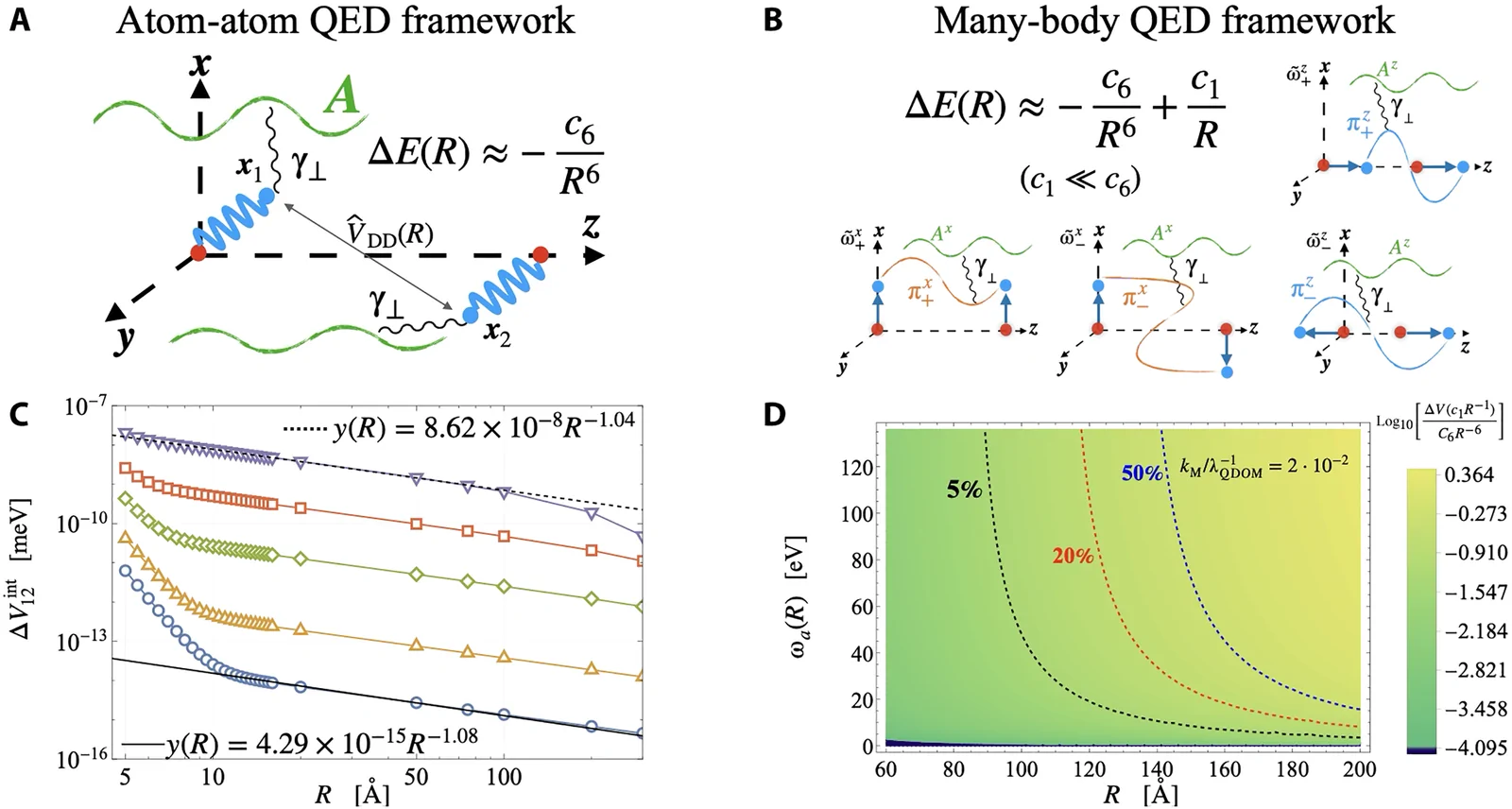
MB-QED mechanism for a repulsive inverse-distance interaction. (**A**) Two QDOs interacting with one another via dipole-dipole coupling ${\hat{V}}_{\text{DD}}$, and with the EMF through the minimal coupling term ${\hat{p}}_{a}\cdot \hat{A}\left(R_{a}\right)$ that results in the exchange of transverse photons $\gamma_{⊥}$. (**B**) QDOs normal modes resulting from diagonalization of the MBD Hamiltonian, i.e. ${\hat{H}}_{\text{MBD}}={\hat{H}}_{\text{QDO}}^{\left(1\right)}+{\hat{H}}_{\text{QDO}}^{\left(2\right)}+{\hat{V}}_{\text{DD}}$ (see the Supplementary Materials for the diagonalization). There are six normal modes, including two longitudinal modes ($\pi_{\pm }^{z}$) and four transverse modes ($\pi_{\pm }^{x}$, and two other modes with $\pi_{\pm }^{y}$ that are not shown here). These modes interact with the EMF, exchanging transverse photons $\gamma_{⊥}$, via ${\hat{H}}_{\text{int}}=-\sum_{L,i}{\hat{\pi }}_{L,i}A_{L,i}$ as in Eq. 5. (**C**) Interaction energy $\Delta V_{12}^{\text{int}}$ defined in Eq. 11 as a function of the inter-QDO distance *R* for different values of the cutoff ratio $k_{M}/\lambda_{\text{QDOM}}^{-1}$, where $\lambda_{\text{QDOM}}={\text{max}}_{a}\sqrt{\hbar /2m\omega_{a}}$ (see main text). The violet triangles, red squares, green diamonds, orange triangles, and blue circles correspond respectively to $k_{M}/\lambda_{\text{QDOM}}^{-1}=2\cdot 10^{-2},\;10^{-2},\;5\cdot 10^{-3},\;2\cdot 10^{-3}$, and 10^−3^. The dashed black line shows the fit $y\left(R\right)=8.62\times 10^{-8}R^{-1.04}$, while the solid black line corresponds to $y\left(R\right)=4.29\times 10^{-15}R^{-1.08}$. Notice that the deviations from the 1/*R* behavior at short range and low frequencies arise from the higher-order terms, particularly the *R*^−9^ term in (*12*), which dominates over 1/*R* for smaller values of the cutoff wave number *k*_M_ (see section S5 for a detailed analysis). (**D**) Contour plot of ${\text{log}}_{10}\left[\Delta V\left(c_{1}R^{-1}\right)/c_{6}R^{-6}\right]$ as a function of QDO frequencies and interatomic distance *R*. This illustrates the crossover between the MB-QED correction and the vdW interaction across spatial and frequency scales.

The well-established atom-atom QED approach, first developed for two interacting atoms by Casimir and Polder (*24*), assumes that atoms are polarizable entities that interact perturbatively with each other and with the EMF (Fig. 1A) (*3*, *5*, *6*). This approach ignores interatomic correlations and high-frequency field modes. This logic underpins the vast majority of EMF force calculations, except for rare case examples where nonperturbative approaches were considered (*25*–*28*).

In realistic molecules and solids, interacting atoms form a correlated electronic system. Interatomic bonding and MB correlations must be taken into account before coupling to the EMF vector potential. Therefore, in contrast to the conventional approach, we focus on interacting matter—incorporating all dipolar Coulomb couplings between the atoms—as our unperturbed baseline system. By first determining the collective energy eigenstates of this correlated matter system, we then apply perturbation theory solely to evaluate the coupling between these collective states and the vacuum EMF. Our formalism reveals a repulsive interaction in the near-zone that scales as *R*^−1^ between neutral atoms, arising directly from the coupling between molecular plasmons and virtual photons.

## RESULTS

### MB-QED framework

We developed a general MB-QED framework for a system of QDOs coupled with the EMF (see the Supplementary Materials for the derivation). However, our main findings can already be explained by the example of two coupled atoms forming molecular plasmons, which we present in what follows. We consider the Hamiltonian describing the QDO system in interaction with the EMF

$${\hat{H}}_{\text{QDO}-\text{QED}}=\sum_{a=1}^{2}{\hat{H}}_{a}+{\hat{V}}_{\text{DD}}\left(R\right)+{\hat{H}}_{\text{EMF}}+{\hat{H}}_{\text{int}}$$ (1)

The term ${\hat{H}}_{a}={|{\hat{p}}_{a}|}^{2}/2m_{a}+m_{a}\omega_{a}^{2}{|{\hat{r}}_{a}|}^{2}/2$ describes the QDO system parametrized by effective mass *m_a_*, charge *q_a_*, and frequency ω*_a_*, and is represented by the canonical operators ${\hat{r}}_{a}=\left({\hat{x}}_{a},{\hat{y}}_{a},{\hat{z}}_{a}\right)$ and ${\hat{p}}_{a}=\left({\hat{p}}_{a,1},{\hat{p}}_{a,2},{\hat{p}}_{a,3}\right)$ with a=1,2, located on the *z* axis at $R_{1}=R_{1}e_{z}$ and $R_{2}=R_{2}e_{z}$ with mutual distance $R=|R_{2}-R_{1}|$. The oscillators are coupled through the dipole-dipole potential

$${\hat{V}}_{\text{DD}}=\frac{q_{1}q_{2}e^{2}}{4{\pi \varepsilon }_{0}R^{3}}\left({\hat{r}}_{1}\cdot {\hat{r}}_{2}-3{\hat{z}}_{1}{\hat{z}}_{2}\right)$$ (2)

The quantum energy levels for each QDO are $E_{a}^{i}=\hbar \omega_{a}^{i}\left(m^{i}+1/2\right)$ where *m^i^* is the quantum number in the *i*-th spatial direction.

The EMF, represented by the vector potential, $\hat{A}$, is considered in Coulomb gauge ∇⋅A=0 and in dipole approximation, i.e., assuming $\hat{A}\left(R_{a}+r_{a}\right)≃\hat{A}\left(R_{a}\right)$. This approximation is taken with respect to the internal coordinate **r***_a_* of each QDO only. It does not remove the dependence of the field on the QDO center **R***_a_*. Therefore, after evaluating the field at **R***_a_*, the plane-wave expansion of $\hat{A}$ still contains the phase factor $e^{ik\cdot R_{a}}$, which encodes the finite separation between different QDO centers and is responsible for the photon-mediated intercenter coupling. In so doing, ${\hat{H}}_{\text{EMF}}$ is the free Hamiltonian of the EMF, which is the sum of the squares of the electric and magnetic fields, while the QDO-EMF interaction term takes the form ${\hat{H}}_{\text{int}}=\sum_{a}\left[-q_{a}{\hat{p}}_{a}\cdot \hat{A}\left(R_{a}\right)/m_{a}+q_{a}^{2}{\hat{A}}^{2}\left(R_{a}\right)/2m_{a}\right]$, where ${\hat{A}}_{j}\left(x\right)=\sum_{k,\lambda }\sqrt{\hbar {\left(2\varepsilon_{0}\mathrm{ckV}\right)}^{-1}}\varepsilon_{j,\lambda }\left(k\right)\left({\hat{a}}_{k\lambda }e^{ik\cdot x}+c.c.\right)$, **k** is the wave vector, $\left\{\varepsilon_{\lambda }\left(k\right)\right\}$ are the polarization vector basis, and ${\hat{a}}_{k\lambda }$ and ${\hat{a}}_{k\lambda }^{†}$ are, respectively, the photon annihilation and creation operators. A key step in treating the EMF is to specify the set of allowed modes, namely, the permitted wave vectors **k** and polarizations **ε**_λ_. The infrared regime is regularized by considering a finite cubic box of size *L* = *V*^1/3^ and considering the limit for L→+∞ once the transition amplitudes between asymptotic states are computed. We regularize the ultraviolet behavior by imposing an upper cutoff *k*_M_ on the wave numbers of the EMF modes. This cutoff is required because the MBD model is an effective theory that reproduces the electric charge-density response of atoms in molecules only down to length scales no shorter than the characteristic QDO length, $\lambda_{\text{QDOM}}={\text{max}}_{a=1,2}\sqrt{\frac{\hbar }{2m_{a}\omega_{a}}}$. Accordingly, we choose $k_{M}=\eta \;\lambda_{\text{QDOM}}^{-1}$, with 0<η≤1. The need for such a cutoff is further supported by the nonrenormalizability of the effective theory defined by the Hamiltonian in Eq. 1. Because the QDO-EMF coupling has negative mass dimension, ultraviolet divergences cannot, in general, be absorbed into a finite set of parameters (*29*). The theory should therefore be understood as an effective description, valid below a cutoff set by the length scale at which the QDO model ceases to resolve the underlying charge-density response.

Within the perturbative scheme adopted here, the diamagnetic **A**^2^ term does not affect the distance-dependent interaction energy at the leading order considered in this work (see section S2). Its first-order contribution gives a local self-energy shift, whereas its second-order contribution is of higher order in the charge than the leading correction generated by the paramagnetic p⋅A coupling. Therefore, to isolate the leading distance-dependent radiative correction, the **A**^2^ term can be omitted consistently within the present approximation. We stress, however, that this omission is not a conceptual requirement: An equivalent formulation can absorb the diamagnetic contribution into dressed photon frequencies through a Bogoliubov transformation (*30*).

We treat the dipole-dipole interaction exactly by diagonalizing the coupled Hamiltonian ${\hat{H}}_{0}={\hat{H}}_{1}+{\hat{H}}_{2}+{\hat{V}}_{\text{DD}}$. This diagonalization defines collective normal modes—molecular plasmons—whose renormalized frequencies ${\tilde{\omega }}_{L,i}$ already include the full instantaneous electronic correlation between the atoms. The transformation from individual to collective operators is realized through the orthonormal change of basis ${\hat{X}}_{L}=\sum_{a=1}^{2}C_{\mathrm{aL}}\;{\hat{r}}_{a}$, ${\hat{\pi }}_{L}=\sum_{a=1}^{2}C_{\mathrm{aL}}\;{\hat{p}}_{a}$, with

$$C_{1+}=C_{2+}=C_{1-}=\frac{1}{\sqrt{2}}11,\;C_{2-}=-\frac{1}{\sqrt{2}}11$$ (3)

In these collective coordinates, the QDO-QED Hamiltonian in Eq. 1 writes

$${\hat{H}}_{\text{MB}-\text{QED}}={\hat{H}}_{\text{MBD}}+{\hat{H}}_{\text{int}}+{\hat{H}}_{\text{EMF}}$$ (4)

where the interaction reads

$${\hat{H}}_{\text{int}}=-\sum_{L,i}{\hat{\pi }}_{L,i}\;{\hat{A}}_{L,i},\;{\hat{A}}_{L,i}=\sum_{a,j}\frac{q_{a}{\left(C_{\mathrm{aL}}\right)}_{\mathrm{ij}}}{\sqrt{m_{a}}}{\hat{A}}_{i}\left(R_{a}\right)$$ (5)

The molecular subsystem assumes a diagonal form

$${\hat{H}}_{\text{MBD}}=\frac{1}{2}\sum_{L=\pm }\sum_{i=1}^{3}\left({\hat{\pi }}_{L,i}^{2}+{\tilde{\omega }}_{L,i}^{2}{\hat{X}}_{L,i}\right)$$ (6)

representing the system of six normal-mode QDOs, each characterized by its corresponding normal-mode frequency ${\tilde{\omega }}_{\left(L,i\right)}$ (Fig. 1B). The ground-state energy of this collective system contains not only the ground-state energy of isolated QDOs (described by $\sum_{a=1}^{2}{\hat{H}}_{a}$), but also the distance-dependent interaction energy that arises from the instantaneous and anisotropic dipole-dipole coupling between them. This interaction energy contains an infinite series of London dispersion and polarization contributions, starting with the leading order *R*^−6^ term (*31*). This implies that the polarizability of the QDO dimer is nonadditive at any separation where the dipole-dipole potential works.

### Perturbative QED energy from collective modes

Within this collective formulation, the leading radiative energy arises from the second-order interaction between each normal mode and the quantized EMF

$$\Delta E^{\text{MB}-\text{QED}}=-\sum_{i,L}\sum_{k\mu }\frac{{\left|\langle {\tilde{1}}_{i,L};1_{k,\mu }|{\hat{H}}_{\text{int}}|0_{i,L};0_{k\mu }\rangle \right|}^{2}}{\hbar \mathrm{ck}+\hbar {\tilde{\omega }}_{i,L}}$$ (7)

A cumbersome calculation (see section S4) recasts the radiative correction above into an intermediate form

$$\begin{array}{c} \Delta E^{\text{MB}-\text{QED}}=-\frac{\hbar }{4\varepsilon_{0}c}\sum_{L,j}\int \frac{\mathrm{dk}\;d\Omega_{k}}{{\left(2\pi \right)}^{3}}\frac{k{\tilde{\omega }}_{\mathrm{Lj}}}{\left(\mathrm{ck}+{\tilde{\omega }}_{\mathrm{Lj}}\right)}\\ \times \sum_{a,b}\sum_{i,\ell }\frac{q_{a}q_{b}}{\sqrt{m_{a}m_{b}}}{\left(C_{\mathrm{aL}}\right)}_{\mathrm{ij}}{\left(C_{\mathrm{bL}}\right)}_{\ell j}P_{i\ell }\left(k\right)\;e^{ik\cdot \left(R_{a}-R_{b}\right)} \end{array}$$ (8)

Note that we have defined $P_{\mathrm{ij}}\left(k\right)≔\sum_{\lambda }\varepsilon_{i,\lambda }\left(k\right)\varepsilon_{j,\lambda }\left(k\right)=\delta_{\mathrm{ij}}-k_{i}k_{j}/k^{2}$. The integral naturally separates into a self-energy part $\Delta U_{a}^{\text{self}}\left(k_{M}\right)$ for *a* = *b* and an interaction part $\Delta V_{12}^{\text{int}}\left(R,k_{M}\right)$ for (a≠b), corresponding respectively to the dressing of each collective mode and the photon-mediated coupling between them.

The self-energy term carries the usual ultraviolet divergence of QED. We choose the renormalization scheme such that the reference configuration is represented by a set of dynamically isolated collective modes, rather than isolated atoms. This choice is the most appropriate to enforce consistency with the MBD Hamiltonian ${\hat{H}}_{\text{MBD}}$ since it naturally preserves its normal-mode structure (see section S4). The reference shift for each mode reads

$$\Delta E_{a}^{\text{free}}\left(k_{M}\right)=-\frac{\hbar }{12\pi^{2}\varepsilon_{0}c}\frac{q_{a}^{2}}{m_{a}}\sum_{L,j}C_{a,L}\int_{0}^{k_{M}}\mathrm{dk}\;\frac{k\;{\tilde{\omega }}_{\mathrm{Lj}}}{\mathrm{ck}}$$ (9)

where $C_{a,L}≔\text{Tr}\left(C_{\mathrm{aL}}^{T}C_{\mathrm{aL}}\right)$.

Subtracting this term from Eq. 8 removes the nonphysical divergence and yields a finite, physically meaningful correction to the self-energy, i.e., $\Delta U_{a}^{\text{self}}\left(k_{M}\right)-\Delta U_{a}^{\text{free}}\left(k_{M}\right)$, which gives

$$\begin{array}{c} \Delta U_{a}^{\text{self},\;\text{ren}}\left(k_{M}\right)=\frac{\hbar }{12\pi^{2}\varepsilon_{0}c^{3}}\frac{q_{a}^{2}}{m_{a}}\\ \times \sum_{L,j}C_{a,L}{\tilde{\omega }}_{\mathrm{Lj}}^{2}\text{ln}\left[\frac{{\mathrm{ck}}_{M}+{\tilde{\omega }}_{\mathrm{Lj}}}{{\tilde{\omega }}_{\mathrm{Lj}}}\right] \end{array}$$ (10)

Although Eq. 10 depends on *R* implicitly through the collective normal-mode frequencies ${\tilde{\omega }}_{\mathrm{Lj}}\left(R\right)$, it does not contain an explicit photon-mediated intercenter phase factor. The latter is instead contained in the finite contribution with a≠b, which carries the explicit spatial factor $e^{ik\cdot \left(R_{a}-R_{b}\right)}$ and defines the effective interaction between collective excitations

$$\begin{array}{c} \Delta V_{12}^{\text{int}}\left(R,k_{M}\right)=-\frac{\hbar }{4\pi^{2}\varepsilon_{0}c}\frac{2\;q_{1}q_{2}}{\sqrt{m_{1}m_{2}}}\\ \times \sum_{j}\int_{0}^{k_{M}}\bar{f}\left(\mathrm{kR}\right)\left[\frac{k\;{\tilde{\omega }}_{+j}}{\mathrm{ck}+{\tilde{\omega }}_{+j}}-\frac{k\;{\tilde{\omega }}_{-j}}{\mathrm{ck}+{\tilde{\omega }}_{-j}}\right]\mathrm{dk} \end{array}$$ (11)

where $\bar{f}=\left(f_{∥}+2f_{⊥}\right)/2$, $f_{⊥}\left(x\right)=\left[x\text{cos}x+\left(x^{2}-1\right)\text{sin}x\right]/x^{3}$, and $f_{∥}\left(x\right)=2\left[\text{sin}x-x\text{cos}x\right]/x^{3}$. This term encapsulates the full radiative coupling between molecular plasmons. All terms are finite, parameter-free, and directly determined by the microscopic parameters of the QDO model.

### The emergence of the inverse-distance interaction

The inverse-distance behavior of the interaction energy $\Delta V_{12}^{\text{int}}\left(R,k_{M}\right)$ emerges when the structure of Eq. 11 is examined in the near-zone regime. In this limit, both the MBD eigenstates and the eigenfrequencies can be perturbatively expanded in powers of the dipole-dipole coupling constant $\gamma =A_{0}/R^{3}$, around the corresponding values for the uncoupled QDOs. Moreover, the geometric functions $f_{∥}\left(\mathrm{kR}\right)$ and $f_{⊥}\left(\mathrm{kR}\right)$ can be expanded in series around τ=kR=0. After expanding the integrand in Eq. 11 to third order in γ and τ, performing the integration over *k*, and restoring the explicit dependence on *R* (see section S5 for further details), one obtains a compact expression that captures the spatial dependence of the QED contribution to the interatomic interaction potential

$$\Delta V_{12,\text{app}}^{\text{int}}\left(R,k_{M}\right)=\Delta V\left[\frac{c_{1}\left(k_{M}\right)}{R}+\frac{c_{7}\left(k_{M}\right)}{R^{7}}+\frac{c_{9}\left(k_{M}\right)}{R^{9}}\right]$$ (12)

where Δ*V* denotes a reference energy scale and where the coefficients are reported in the Supplementary Materials. Adopting atomic units (${\left[c\right]}_{a.u.}=\alpha_{\text{fsc}}^{-1}$), we find

$$\Delta V=\alpha_{\text{fsc}}^{3}E_{h}{\left[E_{M}\right]}_{a.u.}{\left[{\left(A_{0,1}A_{0,2}\right)}^{1/2}\right]}_{a.u.}{\left[{\left(E_{1}E_{2}\right)}^{3/2}\right]}_{a.u.}/\pi$$ (13)

with $E_{M}=\hbar {\mathrm{ck}}_{M}$ being the ultraviolet cutoff for energies, *E*_h_ the Hartree energy scale, and α_fsc_ the fine-structure constant. The coefficients in Eq. 12 generally depend on the ultraviolet cutoff *k*_M_ imposed on the EMF modes, as expected in a regularized quantum field theory. In renormalizable theories, cutoff dependence can be absorbed into a finite set of renormalized parameters, so that physical observables remain independent of the regulator after renormalization. Here, however, the Hamiltonian defines an effective theory with a finite range of validity. In the absence of direct experimental constraints on these quantities, we therefore retain the explicit dependence on the cutoff.

When compared to the explicit numerical solution of Eq. 11, the analytic prediction remains valid over a wide distance range (∼5 to 100 Å) even for relatively large values of *k*_M_. The MB-QED interaction scales with $\alpha_{\text{fsc}}^{3}$, which is reminiscent of the Lamb shift for a single atom. We observe that the only QED contribution that decays slower than the standard *R*^−6^ van der Waals (vdW) interaction is the term $c_{1}\;\Delta V/R$, with $c_{1}\left(k_{M}\right)>0$, leading to a repulsive long-range interaction. This term originates by taking the first order in $\gamma \propto R^{-3}$ and the second order in $\tau \propto R^{2}$ in the series expansion used to derive Eq. 11. This provides an interpretation of the *R*^−1^ term as arising from elementary quanta-exchange processes between QDOs and the EMF.

Within the second-quantized MBD formalism of (*9*), which uses a Bogoliubov transformation between the atomic QDO and the MBD creation/annihilation operators, the eigenstates of the MBD Hamiltonian can be expressed as linear superpositions of free atomic QDO eigenstates, expanded in powers of γ. In this framework, dipole-dipole MBD interactions induce virtual currents in the atomic QDOs, which subsequently couple to the EMF, effectively acting as excited dipolar currents. Our result can thus be interpreted in analogy with previous works on the emergence of long-range interactions between excited dipole states in both classical (*32*) and QED (*33*, *34*).

Our approximate analytical derivation is fully confirmed by explicit numerical evaluation of the radiative integral in Eq. 11. We take the argon dimer as a prototypical example and calculate $\Delta V_{12}^{\text{int}}$ by numerical integration. The results of this numerical computation are reported in Fig. 1C) and compared to the analytic expansion. We find excellent agreement between both methods. This repulsive component becomes particularly relevant in the intermediate regime between the nonretarded and retarded limits, where it can reach up to ∼10% of the total dispersion energy (Fig. 1D) for an argon dimer. The excitation frequency of molecular plasmons controls the overall strength of the MB-QED interaction versus the standard vdW interaction. For example, tightly bound core electrons would contribute more strongly to the MB-QED effect. In contrast, lower excitation frequencies increase the spatial range of the near zone, making the MB-QED interaction longer and more pronounced.

## DISCUSSION

A complete understanding of our result would require working out a nonperturbative approach to field-matter coupling, for example, extending the method proposed by Renne (*26*). MB quantum mechanics shows that one needs at least fourth-order wave functions to reflect the effect of second-order energies on quantum states (*35*). At this stage, we strive to propose possible interpretations of the derived inverse-distance interaction by making an analogy with other 1/*R* terms between point-like particles. The MB-QED effect can be interpreted as arising from a scalar isotropic potential generated by an effective source. Since the only fundamental long-range 1/*R* interactions are electrostatic and gravitational, we assign identical effective charges and masses such that the associated 1/*R* potentials reproduce the QED energy correction. In atomic units, where the Coulomb potential reads $V\left(R\right)=q_{1}q_{2}/R$, the MB-QED interaction takes the form $\Delta V_{12}^{\text{int}}\left(R\right)=\alpha_{\text{fsc}}^{3}\sqrt{A_{0,1}A_{0,2}}\;{\left(E_{1}E_{2}\right)}^{3/2}/\pi R$, where $A_{0,a}$ is the static polarizability (in $a_{0}^{3}$) and $E_{a}=\hbar \omega_{a}$ the QDO excitation energy (in Hartree). By identification with the Coulomb term, we define a per-object effective charge $q_{\text{eff},a}=\alpha_{\text{fsc}}^{3/2}\;A_{0,a}^{1/4}\;E_{a}^{3/4}/\sqrt{\pi }$, such that $\Delta V_{12}^{\text{int}}\left(R\right)=q_{\text{eff},1}\;q_{\text{eff},2}/R.$ Unlike the elementary electron charge, *q*_eff_ is an emergent property of correlated matter; it condenses measurable quantities—polarizability and excitation energies—into an effective coupling to the quantum vacuum. The same 1/*R* law can also be written in a gravitational form, as if the vacuum modes carried an effective imaginary mass renormalized through their coupling to matter. As shown in (*36*, *37*), the static polarizability of QDOs is proportional to the mass $\alpha_{a}^{\text{GKT}}={\mathrm{Cm}}_{a}q_{a}^{2}$ with a=1,2 and where $C=4L^{4}/9\hbar^{2}$ is a constant depending on the characteristic length *L* of the QDO. Now, in Eq. 12, $\Delta V_{12,\text{app}}^{\text{int}}$, the terms Δ*V* is proportional to ${\left(A_{0,1}A_{0,2}\right)}^{1/2}$ and similarly $c_{1}\left(k_{M}\right)={\left(A_{0,1}A_{0,2}\right)}^{1/2}f\left(k_{M}\right)$ where $f(k_{M})$ is a renormalization factor appearing in eq. S79 in the Supplementary Materials. Therefore, we get $\Delta V^{\text{lead}}\left(R\right)=\Delta {\mathrm{Vc}}_{1}/R\propto A_{0,1}A_{0,2}/R$, and using the relation α^GKT^, we obtain $\propto m_{1}m_{2}/R$. All other constants can be collected to form an effective “Newtonian” constant.

In this complementary picture, the prefactor of the interaction plays the role of an emergent source parameter, either “charge-like” or “mass-like,” depending on the chosen analogy. We stress that these emergent quantities are not fundamental charges or masses, but rather effective constructs that capture how collective excitations act as sources of long-range interactions mediated by the vacuum. This dual interpretation underscores the distinctive role of the MB-QED interaction, bridging familiar Coulomb and gravitational forms while remaining rooted in MB-QED. An intriguing question is whether the effective mass and charge scales identified here can be related to the renormalization of mass and charge in interacting quantum field theories. In QED, it is well established that the particle mass and electric charge, together with the coupling constant, are renormalized by interactions, a process often described as the dressing of matter degrees of freedom by the EMF excitations. In close analogy, we conjecture that the effective charge and mass corrections obtained here can be viewed as a renormalization of the QDO parameters (polarizability and characteristic frequency) induced by the dressing of MBD polarization modes with radiative EMF excitations.

There is an alternative semiclassical interpretation of the inverse distance interactions arising from nonlinear contributions to the polarizability of many interacting atoms (*21*). If molecular polarizability were just a sum of atomic polarizabilities, the 1/*R* QED interaction would vanish. The interelectronic coupling makes the polarizability of molecules, materials, and nanostructures highly nonlinear (*38*). Because of this, we expect the inverse distance interaction to be quite general and scale up when complex materials interact. In this context, we conjecture that the inverse-distance interaction can be measured. The force between two modes can be written in atomic units as $F\left(R\right)\approx \frac{2\;\alpha_{\text{fsc}}^{3}}{\pi }\frac{\mu_{1}\mu_{2}\;E_{1}E_{2}}{R^{2}}\frac{E_{h}}{a_{0}}$, where μ is the dipole matrix element (a.u.) and *E* the excitation energy (Ha). The dipole moment is connected to the static polarizability through the standard oscillator-strength relation $\alpha_{0}≃2{|d|}^{2}/E$, with μ≡∣d∣. For an argon dimer ($A_{0}≃11.1$, E≃0.07 Ha), this yields forces of order $10^{-22}-10^{-20}$ N at *R* = 10 to 100 nm, in the zepto-Newton range. For realistic collective excitations with μ≃50−100 D and E≃1−2 eV, the scaling $F\propto \mu_{\text{mode}}^{2}E^{2}/R^{2}$ boosts the signal to $10^{-21}-10^{-19}$ N at separations of 50 to 200 nm. These magnitudes directly overlap with the sensitivity of precision Casimir measurements at micro- and nanometric separations—using torsional oscillators, microcantilevers, or levitated particles—which already operate in the atto- to zepto-Newton regime (*39*, *40*). Likewise, short-range gravity tests with microfabricated resonators and optomechanical sensors are now entering the sub–100-μm domain (*41*), where Casimir backgrounds dominate the signal budget; the distinct 1/*R* scaling provides a clear discriminant from both Casimir ($∼1/R^{7}-1/R^{8}$) and Newtonian ($∼1/R^{2}$) forces, which are searched for in the experiments. The MB-QED contribution gives rise to a force scaling as 1/*R*^2^, placing it within the detection limit of the present Casimir and short-range gravity experiments (atto- to zepto-Newton).

Recent proposals (*42*, *43*) have shown that Bose-Einstein condensates can host emergent long-range forces with 1/*R* character, either mediated by EMFs or arising from collective modes. Our observation of a repulsive 1/*R* potential provides a complementary scenario, suggesting that such effective interactions could compete with or counterbalance the attractive mechanisms discussed in Bose-Einstein condensation contexts. Exploring this interplay experimentally would clarify whether condensates can support stable states governed by a balance of attractive and repulsive long-range forces.

The present approach is based on the QDO model for matter and on a second-order perturbative expansion in the field-matter interaction. As a result, the renormalized masses and self-energies that we derive should be interpreted as effective, model-dependent parameters rather than as universal renormalization constants of QED. In particular, their value depends on the chosen cutoff and on the QDO parametrization, and they cannot be directly identified with the electron mass renormalization of full QED. Furthermore, the restriction to second order means that the validity of our results is limited to the weak-coupling regime, where higher-order photon processes and collective dressing effects can be safely neglected. A natural next step is to pursue a fully nonperturbative treatment of the coupled matter-field system. This would allow one to include photon dressing and mode mixing self-consistently at all orders, without relying on cutoff regularization. Possible avenues include functional-integral techniques, variational approaches to the full field-matter Hamiltonian, or diagrammatic resummations tailored to collective polarization modes. Such methods would clarify the robustness of the 1/*R* term, provide controlled predictions beyond weak coupling, and establish closer connections with renormalization theory in QED.

## MATERIALS AND METHODS

### QDO model

Atoms were modeled as QDOs (*44*), each consisting of a negative Drude particle of charge *q_a_*, mass *m_a_*, and harmonic frequency ω*_a_*, bound to a positive core fixed at position **R***_a_*. The matter Hamiltonian for two QDOs contains the isolated oscillator Hamiltonians and the dipole-dipole Coulomb coupling

$${\hat{H}}_{\text{matter}}=\sum_{a=1}^{2}\left[\frac{{\hat{p}}_{a}^{2}}{2m_{a}}+\frac{1}{2}m_{a}\omega_{a}^{2}{\hat{r}}_{a}^{2}\right]+{\hat{V}}_{\mathrm{DD}}$$ (14)

with

$${\hat{V}}_{\text{DD}}=\frac{q_{1}q_{2}}{4\pi \varepsilon_{0}R^{3}}\left[{\hat{r}}_{1}\cdot {\hat{r}}_{2}-3\frac{\left(R\cdot {\hat{r}}_{1}\right)\left(R\cdot {\hat{r}}_{2}\right)}{R^{2}}\right],\;R=R_{2}-R_{1}$$ (15)

For the dimer geometry used in this work, the intermolecular axis is chosen along *z*, so that the transverse and longitudinal dipole-dipole couplings are $\gamma_{x}=\gamma_{y}=\gamma$ and $\gamma_{z}=-2\gamma$, respectively, with

$$\gamma =\frac{q_{1}q_{2}}{4{\pi \varepsilon }_{0}\sqrt{m_{1}m_{2}}R^{3}}$$ (16)

### Collective normal modes

The dipole-coupled matter Hamiltonian was diagonalized exactly before coupling the system perturbatively to the EMF. We introduced mass-scaled coordinates and momenta

$${\hat{\xi }}_{a}=m_{a}^{1/2}{\hat{r}}_{a},\;{\hat{\pi }}_{a}=m_{a}^{-1/2}{\hat{p}}_{a}$$ (17)

which preserve the canonical commutation relations. In these variables, the matter Hamiltonian separates into three coupled one-dimensional oscillator problems

$${\hat{H}}_{\text{matter}}=\sum_{i=x,y,z}\left[\sum_{a=1}^{2}\left(\frac{{\hat{\pi }}_{\mathrm{ia}}^{2}}{2}+\frac{1}{2}\omega_{a}^{2}{\hat{\xi }}_{\mathrm{ia}}^{2}\right)+\gamma_{i}{\hat{\xi }}_{i1}{\hat{\xi }}_{i2}\right]$$ (18)

Diagonalization gives two collective normal modes for each Cartesian direction, with frequencies

$${\tilde{\omega }}_{i\pm }^{2}=\frac{1}{2}\left[\omega_{1}^{2}+\omega_{2}^{2}\pm \sqrt{{\left(\omega_{1}^{2}-\omega_{2}^{2}\right)}^{2}+4\gamma_{i}^{2}}\right],\;i=x,y,z$$ (19)

For resonant QDOs, the corresponding normal-mode coordinates reduce to the orthonormal symmetric and antisymmetric combinations

$${\hat{\zeta }}_{i\pm }=\frac{{\hat{\xi }}_{i2}\pm {\hat{\xi }}_{i1}}{\sqrt{2}},\;{\hat{\eta }}_{i\pm }=\frac{{\hat{\pi }}_{i2}\pm {\hat{\pi }}_{i1}}{\sqrt{2}}$$ (20)

The resulting MB dispersion Hamiltonian is therefore a set of six collective harmonic modes

$${\hat{H}}_{\text{MBD}}=\sum_{i=x,y,z}\;\sum_{\alpha =\pm }\left[\frac{{\hat{\eta }}_{i\alpha }^{2}}{2}+\frac{1}{2}{\tilde{\omega }}_{i\alpha }^{2}{\hat{\zeta }}_{i\alpha }^{2}\right]$$ (21)

### Quantized EMF and light–matter coupling

The EMF was quantized in Coulomb gauge inside a cubic box of volume *V*. The vector potential was expanded as

$$\hat{A}\left(x\right)=\sum_{k,\lambda }\sqrt{\frac{\hbar }{2\varepsilon_{0}\mathrm{ckV}}}\;e_{\lambda }\left(k\right)\left[{\hat{a}}_{k\lambda }e^{ik\cdot x}+{\hat{a}}_{k\lambda }^{†}e^{-ik\cdot x}\right]$$ (22)

with transverse polarizations satisfying

$$\sum_{\lambda =1}^{2}e_{\lambda ,i}\left(k\right)e_{\lambda ,j}\left(k\right)=\delta_{\mathrm{ij}}-{\hat{k}}_{i}{\hat{k}}_{j}$$ (23)

The dipole approximation was applied only to the internal coordinate of each QDO

$$\hat{A}\left(R_{a}+{\hat{r}}_{a}\right)≃\hat{A}\left(R_{a}\right)$$ (24)

while retaining the phase factor $e^{ik\cdot R_{a}}$, which encodes the finite separation between different QDO centers.

The minimal-coupling interaction contains the paramagnetic $\hat{p}\cdot \hat{A}$ term and the diamagnetic **A**^2^ term. Within the perturbative order considered here, the diamagnetic term gives a local first-order self-energy shift and higher-order charge corrections at second order. Therefore, the leading distance-dependent radiative interaction was obtained from the paramagnetic coupling

$${\hat{H}}_{\text{int}}^{\left(1\right)}=-\sum_{a=1}^{2}\frac{q_{a}}{m_{a}}{\hat{p}}_{a}\cdot \hat{A}\left(R_{a}\right)$$ (25)

After transforming to collective MBD modes, this interaction can be written as

$${\hat{H}}_{\text{int}}^{\left(1\right)}=-\sum_{i,\alpha }{\hat{\eta }}_{i\alpha }{\hat{A}}_{i\alpha }$$ (26)

where ${\hat{A}}_{i\alpha }$ is a collective field operator obtained by weighting the field evaluated at the QDO centers with the normal-mode transformation coefficients.

### Second-order radiative correction

The leading nonvanishing radiative correction was computed using second-order perturbation theory around the product ground state

$$|0\rangle =|0_{\text{MBD}}\rangle ⊗|0_{\text{phot}}\rangle$$ (27)

The relevant intermediate states contain one excitation of a collective MBD mode and one photon. The resulting energy correction has the structure

$$\Delta E^{\left(2\right)}=-\sum_{i,\alpha }\sum_{k,\lambda }\frac{{\left|\langle 1_{i\alpha };1_{k\lambda }|{\hat{H}}_{\text{int}}^{\left(1\right)}|0_{\text{MBD}};0_{\text{phot}}\rangle \right|}^{2}}{\hbar \mathrm{ck}+\hbar {\tilde{\omega }}_{i\alpha }}$$ (28)

After summing over photon polarizations and taking the continuum limit for the field modes, this correction separates into two contributions. Terms with *a* = *b* give self-energy dressing of the collective modes, whereas terms with a≠b contain the explicit spatial phase factor $e^{ik\cdot \left(R_{a}-R_{b}\right)}$ and define the photon-mediated interaction between collective excitations.

The interaction contribution can be written as

$$\Delta V_{12}^{\text{int}}\left(R,k_{M}\right)=-\Delta V\sum_{i=x,y,z}\;\sum_{\alpha =\pm }\int_{0}^{1}C_{i\alpha }^{\left(12\right)}\frac{\bar{k}}{\bar{k}+{\bar{k}}_{\alpha }}F_{i}\left(\bar{k}k_{M}R\right)\;d\bar{k}$$ (29)

where $C_{i\alpha }^{\left(12\right)}$ are the collective-mode correlation factors, $\bar{k}=k/k_{M}$, and

$$F_{i}\left(x\right)=f_{∥}\left(x\right)\delta_{i,z}+f_{⊥}\left(x\right)\left(1-\delta_{i,z}\right)$$ (30)

with

$$f_{⊥}\left(x\right)=\frac{x\text{cos}x+\left(x^{2}-1\right)\text{sin}x}{x^{3}},\;f_{∥}\left(x\right)=\frac{2\left(\text{sin}x-x\text{cos}x\right)}{x^{3}}$$ (31)

### Ultraviolet cutoff and effective-theory interpretation

The QDO model is a finite-resolution effective description of the electronic charge-density response. Accordingly, the electromagnetic modes were restricted to wave numbers below an ultraviolet cutoff

$$k_{M}=\eta \lambda_{\text{QDOM}}^{-1},\;0<\eta \leq 1$$ (32)

where

$$\lambda_{\text{QDOM}}=\underset{a}{\text{max}}\sqrt{\frac{\hbar }{2m_{a}\omega_{a}}}$$ (33)

is the largest characteristic QDO length scale. This cutoff excludes field modes with wavelengths short enough to resolve microscopic charge-density structure beyond the QDO description. The cutoff dependence of the resulting coefficient is therefore retained explicitly and interpreted as part of the effective finite-resolution MB-QED model.

### Near-zone analytical expansion and numerical evaluation

To extract the distance dependence, the integral for $\Delta V_{12}^{\text{int}}\left(R,k_{M}\right)$ was analyzed in the near-zone regime. The collective-mode frequencies and correlations were expanded in the dipole-dipole coupling parameter

$$\gamma =\frac{A_{0}}{R^{3}}$$ (34)

while the angular functions were expanded in powers of

$$\tau =k_{M}R$$ (35)

Keeping the leading terms in this joint expansion yields

$$\Delta V_{12}^{\text{int}}\left(R,k_{M}\right)≃\Delta V\left[\frac{c_{1}\left(k_{M}\right)}{R}+\frac{c_{7}\left(k_{M}\right)}{R^{7}}+\frac{c_{9}\left(k_{M}\right)}{R^{9}}\right]$$ (36)

For the QDO parameters used to model argon, the coefficient *c*_1_(*k*_M_) is positive over the cutoff range considered, giving a repulsive inverse-distance contribution. The analytical expansion was benchmarked against direct numerical integration of the full radiative integral for the argon dimer.

## Supplementary Materials

### This PDF file includes:

Sections S1 to S5 Fig. S1 References
