# Supplementary material for: Repulsive inverse-distance interatomic interaction from many-body quantum electrodynamics
Source: Sci Adv. 2026 Aug 28;12(35):eaee5141. doi: 10.1126/sciadv.aee5141 (PMC13524045; doi:10.1126/sciadv.aee5141)
Supplement: Supplementary file 1 [file sciadv.aee5141_sm.pdf]

Science Advances
AAAS

Supplementary Materials for

Repulsive inverse-distance interatomic interaction from many-body quantum electrodynamics

Loris Di Cairano *et al.*

Corresponding author: Alexandre Tkatchenko, alexandre.tkatchenko@uni.lu

Sci. Adv. **12**, eaee5141 (2026)
DOI: 10.1126/sciadv.aee5141

This PDF file includes:

Sections S1 to S5
Fig. S1
References

1 QDO model for matter system

The quantum Drude oscillator (QDO) is a coarse-grained quantum-mechanical model that describes the electronic response of valence electrons in atoms and molecules (*44*). The model is composed of two pseudo-charges, an infinitely heavy nucleus(–like) positive charge $q_{nucl} = -q$ located at the point $\boldsymbol{R}$, and a negatively charged Drudon q with the position vector $\boldsymbol{x}$, bound to the nucleus via a harmonic potential,

$$\frac{1}{2}m\omega^2|\boldsymbol{x}-\boldsymbol{R}|^2 \; . \tag{S1}$$

The model parameters, m and ω, respectively, denote the mass and the frequency of the QDO. The interaction of the Drude oscillator with the (vacuum) electromagnetic field can be introduced through the minimal coupling principle. However, we first note that we are interested in long-wave phenomena related to dipolar properties of the matter system. Therefore, we assume that the vector potential does not appreciably vary on the characteristic length scale of the matter system. In the other word, we assume that $\boldsymbol{A}(\boldsymbol{x}) \approx \boldsymbol{A}(\boldsymbol{R})$, hence the minimal coupling is given by

$$\boldsymbol{p} \mapsto \boldsymbol{p} - q\boldsymbol{A}(\boldsymbol{R}) \; , \tag{S2}$$

where $\boldsymbol{p}$ is the momentum of the Drudon. Thus, the Hamiltonian of the matter system (modeled by a single QDO) interacting with the free electromagnetic field is given by

$$H = H_{\text{matter}} + H_{\text{field}} + H_{\text{int}} = \frac{1}{2m}\left|\boldsymbol{p} - q\boldsymbol{A}(\boldsymbol{R})\right|^2 + \frac{1}{2}m\omega^2|\boldsymbol{x}-\boldsymbol{R}|^2 + H_{\text{field}} \; . \tag{S3}$$

The first term in the Hamiltonian produces three terms (in the Coulomb gauge) that read

$$\frac{1}{2m}\left|\boldsymbol{p} - q\boldsymbol{A}(\boldsymbol{R})\right|^2 = \frac{1}{2m}\boldsymbol{p}^2 - \frac{q}{m}\boldsymbol{p}\cdot\boldsymbol{A}(\boldsymbol{R}) + \frac{q^2}{2m}\boldsymbol{A}^2 \; , \tag{S4}$$

so that we recognize the interaction terms to be

$$H_{\text{int}} = -\frac{q}{m}\boldsymbol{p}\cdot\boldsymbol{A}(\boldsymbol{R}) + \frac{q^2}{2m}\boldsymbol{A}^2(\boldsymbol{R}) \; . \tag{S5}$$

The quantization of the QDO Hamiltonian is done by following the Dirac's prescription: we promote $(\boldsymbol{p},\boldsymbol{x})$ to quantum mechanical operators satisfying the canonical commutation relation

$$[\hat{x}_{ia}, \hat{p}_{jb}] = i\delta_{ab}\delta_{ij}\hbar. \tag{S6}$$

where the indexes a,b labels respectively the a-th and b-th atomic QDOs and the indexes $i,j=x,y,z$ labels the cartesian coordinates.

The matter–matter interaction for two QDOs is the Coulomb coupling between the charged (pseudo) particles of the two entities. In the dipole approximation, this QDO–QDO interaction is given by

$$\hat{V}_C(\hat{\boldsymbol{x}}_1,\hat{\boldsymbol{x}}_2)\approx\hat{V}_{\mathrm{DD}}(\hat{\boldsymbol{r}}_1,\hat{\boldsymbol{r}}_2,\boldsymbol{R})=\frac{q_1q_2}{4\pi\varepsilon_0R^3}\left[\hat{\boldsymbol{r}}_1\cdot\hat{\boldsymbol{r}}_2-3\frac{(\boldsymbol{R}\cdot\hat{\boldsymbol{r}}_1)(\boldsymbol{R}\cdot\hat{\boldsymbol{r}}_2)}{R^2}\right], \tag{S7}$$

where $\hat{\boldsymbol{r}}_a$ is the a-th atomic Drudon position relative to the atomic center, i.e., $\hat{\boldsymbol{r}}_a=\hat{\boldsymbol{x}}_a-\boldsymbol{R}_a$, and $\boldsymbol{R}=\boldsymbol{R}_2-\boldsymbol{R}_1$ denotes the relative position vector between the two QDO centers. For simplicity, we assume that $\boldsymbol{R}$ is along the z-axis, so $\boldsymbol{R}=R\hat{e}_z$ being $\hat{\boldsymbol{e}}_z$ the unity vector along the z-axis. Therefore, the matter Hamiltonian, including the dipole–dipole Coulomb interaction $\hat{V}_{12}^{\mathrm{dip-dip}}$, can be written as

$$\hat{H}_{\mathrm{matter}}=\sum_{a=1,2}\left[\frac{\hat{\boldsymbol{p}}_a^2}{2m_a}+\frac{1}{2}m_a\omega_a^2\hat{\boldsymbol{r}}_a^2\right]+\frac{q_1q_2}{4\pi\varepsilon_0R^3}\left(\hat{\boldsymbol{r}}_1\cdot\hat{\boldsymbol{r}}_2-3\hat{z}_1\hat{z}_2\right)=\sum_{a=1,2}\frac{\hat{\boldsymbol{p}}_a^2}{2m_a}+(\hat{\boldsymbol{r}}_1,\hat{\boldsymbol{r}}_2)\,\mathbb{V}\begin{pmatrix}\hat{\boldsymbol{r}}_1\\ \hat{\boldsymbol{r}}_2\end{pmatrix}, \tag{S8}$$

where we have introduced the potential energy matrix

$$\mathbb{V}=\begin{pmatrix}\omega_1^2\mathbb{I}_3 & \mathbb{T}(\boldsymbol{R}_{12})\\ \mathbb{T}(\boldsymbol{R}_{12}) & \omega_2^2\mathbb{I}_3\end{pmatrix}, \tag{S9}$$

with $\mathbb{T}(\boldsymbol{R}_{12})$ denoting the dipole–dipole coupling matrix.

1.1 Diagonalization of the matter Hamiltonian

In the case of a quadratically coupled quantum harmonic oscillator, such as for $\hat{H}_{\mathrm{matter}}$, the reduction of the Hamiltonian to its normal form is equivalent to the diagonalization of the symmetric, positive-definite matrix associated with the quadratic potential. We define the set of mass scaled coordinates $\hat{\boldsymbol{\xi}}_a=m^{1/2}\hat{\boldsymbol{r}}_a$ and momenta $\hat{\boldsymbol{\pi}}_a=m_a^{-1/2}\hat{\boldsymbol{p}}_a$. We notice here that the mass scaling transformation reported above preserve the canonical commutation relations (CCR), i.e. $[\hat{\xi}_{ia},\hat{\pi}_{jb}]=i\hbar\delta_{ij}\delta_{ab}$ and $[\hat{\xi}_{ia},\hat{\xi}_{jb}]=[\hat{\pi}_{ia},\hat{\pi}_{jb}]=0$. Moreover, by introducing the coupling parameters

$$\gamma_x=\gamma_y=\gamma=\frac{q_1q_2}{4\pi\varepsilon_0\sqrt{m_1m_2}R^3}\ ,\quad\text{and}\quad\gamma_z=-2\gamma \tag{S10}$$

it is possible to rewrite the matter Hamiltonian $\hat{H}_{\text{matter}}$ as follows

$$\hat{H}_{\text{matter}} = \sum_{i=x,y,z} \left[\sum_{a=1,2} \left(\frac{\hat{\pi}_{ia}^2}{2} + \frac{1}{2}\omega_a^2 \hat{\xi}_{ia}^2 \right) + \gamma_i \hat{\xi}_{i1} \hat{\xi}_{i2} \right] . \tag{S11}$$

The factor 2 in the longitudinal coupling follows directly from the anisotropic tensor structure of the dipole-dipole interaction. For $\mathbf{R} = R\hat{\mathbf{z}}$,

$$\hat{V}_{DD} = \frac{q_1 q_2}{4\pi\varepsilon_0 R^3} \left[\hat{\mathbf{r}}_1 \cdot \hat{\mathbf{r}}_2 - 3\frac{(\mathbf{R}\cdot\hat{\mathbf{r}}_1)(\mathbf{R}\cdot\hat{\mathbf{r}}_2)}{R^2} \right] . \tag{S12}$$

Since

$$\hat{\mathbf{r}}_1 \cdot \hat{\mathbf{r}}_2 = \hat{x}_1\hat{x}_2 + \hat{y}_1\hat{y}_2 + \hat{z}_1\hat{z}_2, \qquad \frac{(\mathbf{R}\cdot\hat{\mathbf{r}}_1)(\mathbf{R}\cdot\hat{\mathbf{r}}_2)}{R^2} = \hat{z}_1\hat{z}_2, \tag{S13}$$

one obtains

$$\hat{V}_{DD} = \frac{q_1 q_2}{4\pi\varepsilon_0 R^3} \left(\hat{x}_1\hat{x}_2 + \hat{y}_1\hat{y}_2 - 2\hat{z}_1\hat{z}_2 \right) . \tag{S14}$$

Hence, the transverse couplings are $\gamma_x = \gamma_y = \gamma$, whereas the longitudinal coupling is $\gamma_z = -2\gamma$.

In matrix representation, it is straightforward to see that Hamiltonian (S11) has the eigenvalues

$$\lambda_{i,\pm} = \frac{1}{4} \left[\omega_1^2 + \omega_2^2 \pm \sqrt{4\gamma_i^2 + (\omega_1^2 - \omega_2^2)^2} \right] \ , \ i = x,y,z \ , \tag{S15}$$

with the corresponding normalized eigenvectors

$$v_{i,\pm} = \left[\frac{\gamma_i^2}{4\gamma_i^2 + [\omega_1^2 - \omega_2^2 \pm \sqrt{4\gamma_i^2 + (\omega_1^2 - \omega_2^2)^2}\,]^2} \right]^{\frac{1}{2}} \begin{pmatrix} \frac{1}{\gamma_i}[\omega_1^2 - \omega_2^2 \pm \sqrt{4\gamma_i^2 + (\omega_1^2 - \omega_2^2)^2}\,] \\ \\ 2 \end{pmatrix} \tag{S16}$$

with $i = x,y,z$.

The diagonalization of the matrix $\mathbb{V}$ allows to define a set of normal modes coordinates $\hat{\xi}_{i\pm}$

$$\zeta_{i+} = \mathscr{N}_{i+}^{(1)} \xi_{i,1} + \mathscr{N}_{i+}^{(2)} \xi_{i,2} \ , \quad \zeta_{i-} = \mathscr{N}_{i-}^{(1)} \xi_{i,1} + \mathscr{N}_{i-}^{(2)} \xi_{i,2} \ , \tag{S17}$$

with

$$\mathscr{N}_{i\pm}^{(1)} = \frac{\mathscr{B}_{i\pm}}{\gamma_i} \left[\omega_1^2 - \omega_2^2 \pm \sqrt{4\gamma_i^2 + (\omega_1^2 - \omega_2^2)^2} \right] \ , \qquad \text{and} \qquad \mathscr{N}_{i\pm}^{(2)} = 2\mathscr{B}_{i\pm} \ , \tag{S18}$$

$$\mathscr{B}_{i\pm} = \left[\frac{\gamma_i^2}{8\gamma_i^2 + 2(\omega_1^2 - \omega_2^2)[\omega_1^2 - \omega_2^2 \pm \sqrt{4\gamma_i^2 + (\omega_1^2 - \omega_2^2)^2}\,]^2} \right]^{\frac{1}{2}} . \tag{S19}$$

In a compact, form we will rewrite the previous change of variables as $\boldsymbol{\zeta} = \mathscr{N}\boldsymbol{\xi}$. The corresponding normal-mode momenta $\eta_{i\pm}$ are defined such that $\eta_{i,a} = \mathscr{N}^{(a)}_{i+}\pi_{i+} + \mathscr{N}^{(a)}_{i-}\pi_{i-}$ (with $a = 1,2$ and $i = x,y,z$). The normal-mode coordinates $\xi_{i\pm}$ and momenta $\pi_{i\pm}$ reduce to the simple expressions $\zeta_{i\pm} = \frac{1}{\sqrt{2}}(\xi_{i,2} \pm \xi_{i,1})$ and $\eta_{i\pm} = \frac{1}{\sqrt{2}}(\pi_{i,2} \pm \pi_{i,1})$ if the original isolated QDOs are resonant. The normal-mode coordinates and momenta can be considered as those of six one-dimensional normal-mode oscillators with characteristic frequencies $\omega^2_{i\pm} = 2\lambda_{i,\pm}$ for $i = x,y,z$. The matter Hamiltonian in Eq.(S11) can be rewritten in the normal form

$$\hat{H}_{\text{matter}} = \sum_{\alpha=\pm}\sum_{i=x,y,z}\frac{\hat{\eta}^2_{i\alpha}}{2} + \frac{1}{2}\omega^2_{i\alpha}\hat{\zeta}^2_{i\alpha}\ , \tag{S20}$$

It is convenient, for further computations, to express such a diagonalization procedure of the MBD Hamiltonian in terms of the CCR-preserving transformation from the creation/annihilation operator algebra of the atomic QDOs

$$\hat{\xi}_{ia} = \sqrt{\frac{\hbar}{2\omega_a}}(\hat{a}^\dagger_{ai} + \hat{a}_{ai}) \qquad \hat{\pi}_{ia} = i\sqrt{\frac{\hbar\omega_a}{2}}(\hat{a}^\dagger_{ai} - \hat{a}_{ai}) \tag{S21}$$

to the creation/annihilation operator algebra of the MBD normal modes

$$\hat{\zeta}_{i\alpha} = \sqrt{\frac{\hbar}{2\omega_{i\alpha}}}(\hat{b}^\dagger_{i\alpha} + \hat{b}_{i\alpha}) \qquad \hat{\eta}_{i\alpha} = i\sqrt{\frac{\hbar\omega_{i\alpha}}{2}}(\hat{b}^\dagger_{i\alpha} - \hat{b}_{i\alpha})\ . \tag{S22}$$

Following the results derived in (*9*) the Bogoliubov transformation between creation/annihilation operator of atomic modes and MBD modes

$$\begin{pmatrix}\hat{\boldsymbol{b}} \\ \hat{\boldsymbol{b}}^\dagger\end{pmatrix} = \begin{pmatrix}X & Y \\ Y & X\end{pmatrix}\begin{pmatrix}\hat{\boldsymbol{a}} \\ \hat{\boldsymbol{a}}^\dagger\end{pmatrix} \tag{S23}$$

where the $X = X(\mathscr{N}, \boldsymbol{\omega}, \tilde{\boldsymbol{\omega}})$ and $Y = Y(\mathscr{N}, \boldsymbol{\omega}, \tilde{\boldsymbol{\omega}})$ are the $3N \times 3N$ defined as

$$X = \frac{1}{2}\left[\widetilde{\Omega}^{1/2}\mathscr{N}\Omega^{-1/2} + \widetilde{\Omega}^{-1/2}\mathscr{N}\Omega^{1/2}\right] \qquad Y = \frac{1}{2}\left[\widetilde{\Omega}^{1/2}\mathscr{N}\Omega^{-1/2} - \widetilde{\Omega}^{-1/2}\mathscr{N}\Omega^{1/2}\right] \tag{S24}$$

where $\widetilde{\Omega} = \text{diag}\{\omega_{x,+}, \omega_{x,-}, ...\}$ is the diagonal matrix of normal modes eigenfrequencies and $\Omega = \text{diag}\{\omega_{x,a}, \omega_{x,a}, ...\}$ is the diagonal matrix of atomic QDOs eigenfrequencies.

These matrices allows to define the transformation for the non-dimensionalized quantities, i.e.

$$\begin{aligned}\hat{\xi}_{ia} &= \sqrt{\frac{\hbar}{2\omega_{ia}}}(\hat{a}^\dagger_{ia} + \hat{a}_{ia}) = \sum_{\alpha=\pm}\sqrt{\frac{\hbar}{2\omega_{ia}}}\mathscr{M}^{(a)}_{i\alpha}(\hat{b}^\dagger_{i\alpha} + \hat{b}_{i\alpha}) \\ \hat{\pi}_{a_i} &= i\sqrt{\frac{\hbar\omega_{ia}}{2}}(\hat{a}^\dagger_{a_i} - \hat{a}_{a_i}) = i\sqrt{\frac{\hbar\omega_{ia}}{2}}\sum_{\alpha=\pm}\mathscr{S}^{(a)}_{i\alpha}(\hat{b}^\dagger_{\alpha} - \hat{b}_{\alpha})\end{aligned} \tag{S25}$$

where $\mathscr{M}^{(a)} = X^T - Y^T$ and $\mathscr{S} = X^T + Y^T$. The eigenstates of the MBD Hamiltonian constitute the reference basis set to express the matter state and they are expressed as

$$|\boldsymbol{n}\rangle = \bigotimes_{i=1,2,3} \bigotimes_{\alpha=\pm} |n_{i\alpha}\rangle \qquad E_{\text{matter}} = \sum_{\alpha=\pm} \sum_{i=x,y,z} \hbar\omega_{i\alpha} \left(n_{i\alpha} + \frac{1}{2} \right) . \tag{S26}$$

where $\boldsymbol{n} \in \mathbb{N}^6$ is the label of MBD eigenstate and $n_{i\alpha} \in \mathbb{N}$ is the occupation number of the MBD model labeled by the indexes (i, α).

2 Quantum electromagnetic field

The free electromagnetic field is quantized in a cubic box of volume V, providing at the same time the boundary conditions for the Maxwell equations and the infrared regularization. Within the box a natural basis set is constituted by plane waves with wavevector $\boldsymbol{k}$. The polarizations are determined by the unit vectors $\boldsymbol{e}_\lambda(\boldsymbol{k})$ with $\lambda = 1, 2$ satisfying the following completeness relation

$$\sum_{\lambda=1,2} e_i(\boldsymbol{k}, \lambda) e_j(\boldsymbol{k}, \lambda) = \delta_{ij} - \hat{k}_i \hat{k}_j \, . \tag{S27}$$

Such a physical picture is equivalent to the Fourier expansion of the free field in terms of the plane waves solving the source-free Maxwell equations. Hence, the field $\boldsymbol{A}$ is quantized in the same way an oscillator is, *i.e.* promoting the Fourier expansion coefficients of each mode to quantum mechanical operators that obey canonical commutation relations

$$[\hat{a}_\lambda(\boldsymbol{k}), \hat{a}^\dagger_{\lambda'}(\boldsymbol{k}')] = \delta_{\boldsymbol{k}\boldsymbol{k}'} \delta_{\lambda\lambda'} \qquad [\hat{a}_\lambda(\boldsymbol{k}), \hat{a}_{\lambda'}(\boldsymbol{k}')] = [\hat{a}^\dagger_\lambda(\boldsymbol{k}), \hat{a}^\dagger_{\lambda'}(\boldsymbol{k}')] = 0 \tag{S28}$$

with $\hat{a}^\dagger_\lambda(\boldsymbol{k})$ and $\hat{a}_\lambda(\boldsymbol{k})$ indicating creation and annihilation operators of the photon in the mode $(\boldsymbol{k}, \lambda)$. Therefore, the quantized field $\boldsymbol{A}$ is given by

$$\hat{\boldsymbol{A}}(\boldsymbol{x}, t) = \sum_{\boldsymbol{k} \in \mathscr{S}(V, k_{\text{M}})} \sum_{\lambda=1}^{2} \sqrt{\frac{\hbar}{2\varepsilon_0 ckV}} \left[\boldsymbol{e}(\boldsymbol{k}, \lambda) \hat{a}_\lambda(\boldsymbol{k}) e^{i\boldsymbol{k}\cdot\boldsymbol{x}} + \boldsymbol{e}(\boldsymbol{k}, \lambda) \hat{a}^\dagger_\lambda(\boldsymbol{k}) e^{-i\boldsymbol{k}\cdot\boldsymbol{x}} \right] , \tag{S29}$$

where $\mathscr{S}(V, k_{\text{M}}) = \{\boldsymbol{k} \in \mathbb{R}^3 \mid \boldsymbol{\eta} \in \mathbb{Z} \wedge \boldsymbol{k} = 2\pi\boldsymbol{\eta}/\sqrt[3]{V} \wedge |\boldsymbol{k}| \le k_M\}$ is the integration domain.

By using the mode-decomposition of the vector field presented in Eq.(S29), the Hamiltonian of the free EM field can be rewritten as

$$\hat{H}_{\text{field}} = \sum_{\boldsymbol{k} \in \mathscr{S}(V, k_{\text{M}})} \sum_{\lambda=1}^{2} \hbar ck \left(\hat{n}_{\boldsymbol{k},\lambda} + \frac{1}{2} \right) \tag{S30}$$

where $\hat{n}_{\boldsymbol{k},\lambda}$ is the number operators of the photons in the mode $(\boldsymbol{k},\lambda)$. Adding a second QDO to the matter system introduces new terms to the Hamiltonian (S3), accounting for additional matter–matter and matter–field interactions.

Introduction of a second QDO does not change the Hamiltonian of the free field, but it adds two new matter–field terms that take into account the interaction of the second QDO with the vacuum field, hence

$$\hat{H}_{\text{int}} = \sum_{a=1,2} \left[-\frac{q_a}{m_a} \hat{\boldsymbol{p}}_a \cdot \hat{\boldsymbol{A}}(\boldsymbol{R}_a) + \frac{q_a^2}{2m_a} \hat{\boldsymbol{A}}^2(\boldsymbol{R}_a) \right] . \tag{S31}$$

2.1 Diamagnetic contribution in perturbation theory

In the perturbative framework adopted below, we employ as a basis the tensor product between the Hilbert space of MBD modes and the Fock basis for the photon field. The unperturbed ground state is

$$|GS\rangle = |0_{\text{MBD}}\rangle \otimes |0_{\text{phot}}\rangle, \tag{S32}$$

with

$$\widehat{a}_\lambda(\boldsymbol{k})|0_{\text{phot}}\rangle = 0, \qquad \widehat{b}_{ia}|0_{\text{MBD}}\rangle = 0.$$

The diamagnetic term in the minimal-coupling Hamiltonian reads

$$\widehat{H}_a^{(2)} = \frac{q_a^2}{2m_a} \widehat{\boldsymbol{A}}(\boldsymbol{R}_a)^2, \tag{S33}$$

where the vector potential operator in the Coulomb gauge is expanded as

$$\widehat{A}_i(\boldsymbol{x}) = \sum_{\boldsymbol{k},\lambda} \sqrt{\frac{\hbar}{2\varepsilon_0 ckV}}\, \varepsilon_\lambda^i(\boldsymbol{k}) \Big(\widehat{a}_\lambda(\boldsymbol{k}) e^{i\boldsymbol{k}\cdot\boldsymbol{x}} + \widehat{a}_\lambda^\dagger(\boldsymbol{k}) e^{-i\boldsymbol{k}\cdot\boldsymbol{x}} \Big). \tag{S34}$$

First-order contribution. The first-order correction to the ground-state energy is

$$\Delta E_{\boldsymbol{A}^2}^{(1)} = \langle GS|\widehat{H}^{(2)}|GS\rangle = \sum_a \frac{q_a^2}{2m_a} \langle 0_{\text{phot}}|\widehat{\boldsymbol{A}}(\boldsymbol{R}_a)^2|0_{\text{phot}}\rangle. \tag{S35}$$

Expanding $\widehat{\boldsymbol{A}}(\boldsymbol{R}_a)^2 = \sum_i \widehat{A}_i(\boldsymbol{R}_a)\widehat{A}_i(\boldsymbol{R}_a)$ and taking the vacuum expectation value, only the contractions $\langle 0|\widehat{a}_\lambda(\boldsymbol{k})\widehat{a}_{\lambda'}^\dagger(\boldsymbol{k}')|0\rangle = \delta_{\boldsymbol{k}\boldsymbol{k}'}\delta_{\lambda\lambda'}$ survive. Thus,

$$\langle 0_{\text{phot}}|\widehat{\boldsymbol{A}}(\boldsymbol{R}_a)^2|0_{\text{phot}}\rangle = \sum_{\boldsymbol{k},\lambda} \frac{\hbar}{2\varepsilon_0 ckV}, \tag{S36}$$

and therefore

$$\Delta E^{(1)}_{\boldsymbol{A}^2} = \sum_a \frac{q_a^2}{2m_a} \sum_{\boldsymbol{k},\lambda} \frac{\hbar}{2\varepsilon_0 ckV}. \tag{S37}$$

This contribution depends only on local quantities (q_a, m_a) and is independent of the interatomic distance R. It represents a purely local self-energy shift, and thus it does not contribute to the interaction energy between QDOs.

Second-order contribution. At second order, the diamagnetic term yields

$$\Delta E^{(2)}_{\boldsymbol{A}^2} = -\sum_{n \neq GS} \frac{|\langle n|\widehat{H}^{(2)}|GS\rangle|^2}{E_n - E_{GS}}, \tag{S38}$$

where $|n\rangle$ runs over all excited photon states of the Fock space,

$$|n\rangle = |0_{\text{MBD}}\rangle \otimes \sum_{\boldsymbol{k},\lambda} |n_{\boldsymbol{k}\lambda}\rangle_{\text{phot}}.$$

In principle, all multiphoton states $|2_{\boldsymbol{k}\lambda}; 2_{\boldsymbol{k}'\lambda'}\rangle$, $|3_{\boldsymbol{k}\lambda}; 3_{\boldsymbol{k}'\lambda'}\rangle$, etc. should be included. However, to identify the dependence on the charge q, it suffices to consider the lowest-order non-vanishing process, in which $\widehat{\boldsymbol{A}}^2$ creates two photons from the vacuum through the term $\widehat{a}^\dagger \widehat{a}^\dagger$. The relevant matrix element is therefore

$$\langle n|\widehat{H}^{(2)}|GS\rangle \sim \sum_a \frac{q_a^2}{2m_a} \langle 2_{\boldsymbol{k}\lambda}; 2_{\boldsymbol{k}'\lambda'}|\widehat{\boldsymbol{A}}(\boldsymbol{R}_a)^2|0_{\text{phot}}\rangle. \tag{S39}$$

From the mode expansion, this element is proportional to

$$\langle n|\widehat{H}^{(2)}|GS\rangle \propto \frac{q_a^2}{m_a} \frac{\hbar}{\varepsilon_0 c\sqrt{kk'}V}. \tag{S40}$$

The corresponding squared amplitude scales as

$$|\langle n|\widehat{H}^{(2)}|GS\rangle|^2 \propto \left(\frac{q_a^2}{m_a}\right)^2 \left(\frac{\hbar}{\varepsilon_0 c}\right)^2 \frac{1}{kk'V^2}. \tag{S41}$$

Since the energy denominator in Eq. (S38) scales as $E_n - E_{GS} \sim \hbar c(k + k')$, we obtain the scaling behavior

$$\Delta E^{(2)}_{\boldsymbol{A}^2} \propto \left(\frac{q_a^2}{m_a}\right)^2 \sim O(q^4). \tag{S42}$$

In conclusion, the $\widehat{\boldsymbol{A}}^2$ term contributes at first order with a local, distance-independent energy shift, and at second order with terms of order $O(q^4)$. Both effects are of higher order compared to the leading $O(q^2)$ correction arising from the $\widehat{\boldsymbol{p}}_a \cdot \widehat{\boldsymbol{A}}$ coupling. Therefore, within the present perturbative framework, the $\widehat{\boldsymbol{A}}^2$ term can be safely neglected when evaluating the interaction energy between QDOs. The extended calculation of $\Delta E^{(2)}_{\boldsymbol{A}^2}$ will be performed in Sec. 5.

3 Second-order perturbation to the matter–field system

We use second-order perturbation theory to obtain the energy shift due to the QDOs–field interactions. First, we consider the first term of H_{int} that is linear in $\boldsymbol{A}$, and rewrite it in terms of the normal-mode momentum operators,

$$\begin{aligned}
\hat{H}^{(1)}_{\text{int}} &= -\sum_{a=1,2}\frac{q_a}{m_a}\hat{\boldsymbol{p}}_a\cdot\hat{\boldsymbol{A}}(\boldsymbol{R}_a) = -\sum_{a=1,2}\sum_{i=x,y,z}\frac{q_a}{\sqrt{m_a}}\hat{\pi}_{ia}\hat{A}_i(\boldsymbol{R}_a) = \\
&= -i\sum_{a=1,2}\sum_{\alpha=\pm}\sum_{i=x,y,z}\sum_{\boldsymbol{k}\in\mathscr{S}(V,k_{\text{M}})}\sum_{\lambda=1}^{2}\sqrt{\frac{\hbar}{2\varepsilon_0 ckV}}\frac{q_a}{\sqrt{m_a}}\sqrt{\frac{\hbar\omega_a}{2}}\mathscr{M}^{(a)}_{i\alpha}\,(\hat{b}^{\dagger}_{ia}-\hat{b}_{ia}) \\
&\times\left[e_i(\boldsymbol{k},\lambda)\hat{a}_{\lambda}(\boldsymbol{k})e^{i\boldsymbol{k}\cdot\boldsymbol{R}_a}+e^{*}_i(\boldsymbol{k},\lambda)\hat{a}^{\dagger}_{\lambda}(\boldsymbol{k})e^{-i\boldsymbol{k}\cdot\boldsymbol{R}_a}\right].
\end{aligned} \tag{S43}$$

The first-order correction to ground state energy are null as

$$\langle 0;\boldsymbol{0}_{\boldsymbol{k},\lambda}|\hat{H}^{(1)}_{\text{int}}|0;\boldsymbol{0}_{\boldsymbol{k},\lambda}\rangle = 0 \tag{S44}$$

due to selection rules, i.e. $\langle 0|\hat{\eta}_{i\alpha}|0\rangle = 0$ and $\langle\boldsymbol{0}_{\boldsymbol{k},\lambda}|\hat{a}_{\lambda}(\boldsymbol{k})|\boldsymbol{0}_{\boldsymbol{k},\lambda}\rangle = \langle\boldsymbol{0}_{\boldsymbol{k},\lambda}|\hat{a}^{\dagger}_{\lambda}(\boldsymbol{k})|\boldsymbol{0}_{\boldsymbol{k},\lambda}\rangle = 0$. Therefore, the first non-zero correction is expected to be at the second order

$$\Delta E^{(pA,2)} = \sum_{I\neq 0}\frac{\langle 0|\hat{H}^{(1)}_{\text{int}}|I\rangle\langle I|\hat{H}^{(1)}_{\text{int}}|0\rangle}{E_0-E_I}\,, \tag{S45}$$

where, due to selection rules, $|I\rangle = |1_{i\alpha};\boldsymbol{1}_{\boldsymbol{k},\lambda}\rangle$ is a state with a single exciton in the collective MBD mode $(i\alpha)$. By using the second quantized formalism for the matter system to compute the expectation values, one obtains

$$\langle 0|i(\hat{b}^{\dagger}_{i\alpha}-\hat{b}_{i\alpha})|1_{i\beta}\rangle\langle 1_{i\beta}|i(\hat{b}^{\dagger}_{i\alpha}-\hat{b}_{i\alpha})|0\rangle = \|\langle 0|i(\hat{b}^{\dagger}_{i\alpha}-\hat{b}_{i\alpha})|1_{i\beta}\rangle\|^2 = \delta_{\alpha\beta} \tag{S46}$$

so that after a straightforward computation

$$\begin{aligned}
\Delta E^{(pA,2)} &= -\sum_{a,a'=1}^{2}\sum_{\alpha=\pm}\sum_{i=x,y,z}\sum_{\boldsymbol{k}\in\mathscr{S}(V,k_{\text{M}})}\sum_{\lambda=1}^{2}\left(\frac{\hbar}{2\varepsilon_0 ckV}\right)\left(\frac{q_j q_{j'}}{\sqrt{m_j m_{j'}}}\right)\mathscr{M}^{(a)}_{i\alpha}\mathscr{M}^{(a')}_{i\alpha}\left(\frac{\hbar\sqrt{\omega_{ia}\omega_{ia'}}}{2}\right) \\
&\times e_i(\boldsymbol{k},\lambda)e^{*}_i(\boldsymbol{k},\lambda)\frac{e^{i\boldsymbol{k}\cdot(\boldsymbol{R}_j-\boldsymbol{R}_{j'})}}{\hbar(\omega_{k\lambda}+\omega_{i\zeta})}\,.
\end{aligned} \tag{S47}$$

To simplify we introduce the normalized QDOs (self-)correlation $\mathscr{C}^{(aa')}_{i\alpha} = \mathscr{M}^{(a)}_{i\alpha}\mathscr{M}^{(a')}_{i\alpha}$ mediated by the MBD mode (i,α) and the non-dimensionalized electric charges $Z_j = q_j/e$. Moreover, we

will consider the continuous limit for the EM field

$$\frac{1}{V}\sum_{k} \longrightarrow \int \frac{d^3k}{8\pi^3} \tag{S48}$$

, and after transforming k-integral into spherical coordinates and using the completeness relation for the polarizations $\boldsymbol{e}(\boldsymbol{k},\lambda)$ one obtain

$$\Delta E^{(pA,2)} = -\alpha_{\text{fsc}} \sum_{a,a'=1}^{2} \sum_{\alpha=\pm} \sum_{i=x,y,z} \frac{\hbar^2}{8\pi^2} \frac{Z_a Z_{a'}}{\sqrt{m_a m_{a'}}} \mathscr{C}_{i\alpha}^{(aa')} \int_0^{k_M} \frac{\sqrt{\omega_a \omega_{a'}}\, k\, dk}{ck+\omega_{i\alpha}} \int_{\mathbb{S}^2} (1-\hat{k}_i\hat{k}_i) e^{i\boldsymbol{k}\cdot\boldsymbol{R}_{aa'}} d\Omega \,. \tag{S49}$$

where $\alpha_{\text{fsc}} = e^2/(4\pi\varepsilon_0\hbar c)$ is the fine structure constant and $\mathbb{S}^2$ is the (unitary) 2-sphere.

The first sum in expression Eq.(S49) can be split in two terms. One can be interpreted as a self-energy of the single atomic QDOs corrections $j = j'$

$$\Delta U_a^{\text{self}} = -\alpha_{\text{fsc}} \sum_{\alpha=\pm} \sum_{i=x,y,z} \frac{E_a}{8\pi^2} \frac{Z_a^2}{m_a c^2} \mathscr{C}_{i\alpha}^{(aa)} \int_0^{E_M} \frac{E\mathrm{d}E}{E+\hbar\omega_{i\alpha}} \int_{\mathbb{S}^2} (1-\hat{k}_i\hat{k}_i) d\Omega \,. \tag{S50}$$

where $E = \hbar ck$ and $E_a = \hbar\omega_a$ By using the completness relations for the polarization of the EM field

$$\int_{\mathbb{S}^2} (\delta_{ij} - \hat{k}_i\hat{k}_j)\mathrm{d}\Omega = \frac{8\pi}{3}\delta_{ij} \tag{S51}$$

and after few algebraic manipulations the expression and using Bethe's renormalization scheme one obtains

$$\Delta U_a^{\text{self}} = \frac{\alpha_{\text{fsc}}}{3\pi} \frac{Q_a^2 E_a}{c^2} \sum_{\alpha=1}^{3N} \sum_{i=x,y,z} \mathscr{C}_{i\alpha}^{(aa)} (\hbar\omega_{i\alpha}) \int_0^{E_M} \frac{\mathrm{d}E}{E+\hbar\omega_{i\alpha}} \,. \tag{S52}$$

where for convenience we have introduced the mass reduced charges $Q_a = Z_a/m_a^{1/2}$.

The other contribution, with $j \neq j'$, corresponds to the corrections to interatomic interaction energy, i.e. inversion symmetry $\boldsymbol{k} \longrightarrow -\boldsymbol{k}$ the angular integral can be rewritten as

$$\int_{\mathbb{S}^2} (1-\hat{k}_i\hat{k}_i) e^{i\boldsymbol{k}\cdot\boldsymbol{R}_{aa'}} d\Omega = \frac{1}{2}\int_{\mathbb{S}^2} (1-\hat{k}_i\hat{k}_i)\left(e^{i\boldsymbol{k}\cdot\boldsymbol{R}_{aa'}} + e^{-i\boldsymbol{k}\cdot\boldsymbol{R}_{aa'}}\right) d\Omega = \int_{\mathbb{S}^2} (1-\hat{k}_i\hat{k}_i)\cos(\boldsymbol{k}\cdot\boldsymbol{R}_{aa'}) d\Omega \tag{S53}$$

so that the full expression can be rewritten as

$$\Delta V_{12}^{\text{int}} = -\alpha_{\text{fsc}} \sum_{\alpha=\pm} \sum_{i=x,y,z} \frac{\hbar^2}{4\pi^2} \frac{Z_1 Z_2 \sqrt{\omega_{i1}\omega_{i2}}}{\sqrt{m_1 m_2} c^2} \mathscr{C}_{i\alpha}^{(12)} \int_0^{E_M} \frac{E\, dE}{ck+\omega_{i\alpha}} \int_{\mathbb{S}^2} (1-\hat{k}_i\hat{k}_i) \cos(\boldsymbol{k}\cdot\boldsymbol{R}_{12})\, d\Omega \,. \tag{S54}$$

Following our conventions on the geometry of the QDOs dimer, the angular integral reads

$$\int_{\mathbb{S}^2} (1 - \hat{k}_i \hat{k}_i) \cos(\boldsymbol{k} \cdot \boldsymbol{R}_{aa'}) \, d\Omega = \frac{4\pi}{3} \left[f_{\parallel}(kR_{aa'}) \delta_{i,z} + f_{\perp}(kR_{aa'})(1 - \delta_{i,z}) \right] \tag{S55}$$

where we have introduced the special functions

$$f_{\perp}(x) = \frac{x\cos(x) + (x^2 - 1)\sin(x)}{x^3} \tag{S56}$$

$$f_{\parallel}(x) = \frac{2\left[\sin(x) - x\cos(x)\right]}{x^3}, \tag{S57}$$

Finally we obtain the expression

$$\Delta V_{12}^{\text{int}} = -\frac{\alpha_{\text{fsc}}}{\pi} \frac{Q_1 Q_2 \sqrt{E_1 E_2}}{c^2} \sum_{\alpha=1}^{3N} \sum_{i=x,y,z} \mathscr{C}_{i\alpha}^{(12)} \int_0^{E_M} \frac{E}{E + \hbar\omega_{i\alpha}} \left[f_{\parallel}(kR_{12}) \delta_{i,z} + (1 - \delta_{i,z}) f_{\perp}(kR_{12})) \right] \mathrm{d}E \tag{S58}$$

4 Analysis of the homoatomic dimer

For the further analysis, it is convenient to rexpress the integral in Eq.(S58) as a non-dimensionalized integral

$$\Delta V_{12}^{\text{int}} = -\Delta V \sum_{\alpha=\pm} \sum_{i=x,y,z} \int_0^1 \mathscr{C}_{i\alpha}^{(12)} \frac{\bar{k}}{\bar{k} + \bar{k}_\alpha} \mathscr{F}_i(\bar{k}\bar{R}) \mathrm{d}\bar{k} \tag{S59}$$

where

$$\mathscr{F}_i(x) = f_{\parallel}(x)\delta_{i,z} + f_{\perp}(x)\,(1 - \delta_{i,z}) \tag{S60}$$

and $\bar{k} = k/k_M$, $\bar{R} = k_M R$, and $\bar{k}_\alpha = E_\alpha/(\hbar c k_M)$. With such substitutions, the integral is adimensional, and the energy scale reads

$$\text{SI units} \qquad \Delta V = \alpha_{\text{fsc}} \frac{E_M}{\pi} \frac{Q_1 Q_2 (E_1 E_2)^{1/2}}{c^2} \tag{S61}$$

$$\text{natural units} \qquad \Delta V = \alpha_{\text{fsc}} \frac{E_M Q_1 Q_2 (E_1 E_2)^{1/2}}{\pi} \tag{S62}$$

$$\text{atomic units} \qquad \Delta V = \alpha_{\text{fsc}}^3 E_h \frac{[E_M]_{a.u.} [(\mathscr{A}_{0,1} \mathscr{A}_{0,2})^{1/2}]_{a.u.} [(E_1 E_2)^{3/2}]_{a.u.}}{\pi} \tag{S63}$$

where $E_M = \hbar c k_M$we have used that in atomic units $[c]_{a.u.} = \alpha_{\text{fsc}}^{-1}$, $Q_a = Z_a/m_a$ is the mass reduced charge, and we have introduced the Hartree energy scale E_h.

In this case the previous expression can be further simplified by introducing the characteristic

QDO wavenumber $\bar{k}_\alpha = [\omega_\alpha/(ck_M)]\,\sigma_\alpha(\gamma) = \bar{k}_Q\sigma_\alpha(\gamma)$ where we have introduced the parameters $\gamma = \mathscr{A}_0/R_{12}^3$ and $\tau = R_{12}k_M$, i.e.

$$\Delta V_{12}^{\text{int}} = -\Delta V \sum_{\alpha=\pm}\sum_{i=x,y,z}\int_0^1 \mathscr{C}_{i\alpha}^{(12)}(\gamma)\mathscr{F}_i(\bar{k}\tau)\frac{\bar{k}\mathrm{d}\bar{k}}{\bar{k}+\bar{k}_Q(\eta)\sigma_\alpha(\gamma)} = -\Delta V\int_0^1 \mathscr{I}(\bar{k};\gamma,\tau,\eta)\mathrm{d}\bar{k}\ . \quad \text{(S64)}$$

In order to provide an analytic approximation, we proceed by approximating the integrand in equation (S64). The $\bar{k}_Q(\eta) = \omega_a/(ck_M(\eta))$ where we used the previous definition of k_M. For the Argon homodimer

$$\bar{k}_Q(\eta) = \omega_a\lambda_{\text{QDO,max}}/(\eta c) = 2.52\times 10^{-3}/\eta\ .$$

The correlation matrix and the MBD eigenfrequencies reads as a function of the γ parameter

$$\mathscr{C}_{z\pm}^{(12)} = \frac{1}{2}\sqrt{1\pm\gamma}\,[\delta_{ab}\mp(1-\delta_{ab})] \quad \text{(S65)}$$

$$\mathscr{C}_{i\pm}^{(12)} = \frac{1}{2}\sqrt{1\pm\gamma}\,[\delta_{ab}\mp(1-\delta_{ab})]\,(1-\delta_{i,z}) \quad \text{(S66)}$$

$$\omega_{z\pm} = \omega\sqrt{1\pm2\gamma} \quad \text{(S67)}$$

$$\tilde{\omega}_{i\pm} = \omega\sqrt{1\pm\gamma} \qquad i=x,y \quad \text{(S68)}$$

The exact expression of the integrand reads

$$\mathscr{I}(\bar{k};\gamma,\tau,\eta) = \frac{1}{\tau^3\bar{k}^2}\Big\{\ \frac{\sqrt{\gamma+1}\left[\left(\tau^2\bar{k}^2-1\right)\sin(\tau\bar{k})+\tau\bar{k}\cos(\tau\bar{k})\right]}{\sqrt{\gamma+1}\,\bar{k}_Q(\eta)+\bar{k}} + \frac{\sqrt{2\gamma+1}\left[\tau\bar{k}\cos(\tau\bar{k})-\sin(\tau\bar{k})\right]}{\sqrt{2\gamma+1}\,\bar{k}_Q(\eta)+\bar{k}} \quad \text{(S69)}$$

$$+\frac{\sqrt{1-2\gamma}\left[\sin(\tau\bar{k})-\tau\bar{k}\cos(\tau\bar{k})\right]}{\sqrt{1-2\gamma}\bar{k}_Q(\eta)+\bar{k}} + \frac{\sqrt{1-\gamma}\left[\sin(\tau\bar{k})-\tau\bar{k}\left(\tau\bar{k}\sin(\tau\bar{k})+\cos(\tau\bar{k})\right)\right]}{\sqrt{1-\gamma}\bar{k}_Q(\eta)+\bar{k}}\ \Big\}\ . \quad \text{(S70)}$$

The full expression derived above is rather cumbersome, and obtaining a more transparent analytical form as a function of the intermolecular distance requires a series of simplifications. To this end, we first observe that for the Argon homodimer, the parameter γ can be approximated as $\gamma = 11.1/[R_{12}]^3_{\text{a.u.}}$. This implies that in the short-range (strongly interacting) regime, one has $\gamma|_{R_{12}=5\,\text{Å}} \approx 1.3\times10^{-2}$, whereas in the long-range (weakly interacting) regime, $\gamma|_{R_{12}=100\,\text{Å}} \approx 1.6\times10^{-6}$. These values justify a perturbative treatment based on an expansion in small γ. However, a major challenge remains due to the presence of trigonometric functions of the variable $\bar{k}$ in the integrand, which complicates analytical handling. A potential reduction in complexity can be

achieved by performing a Taylor expansion in the limit $\tau \to 0$. However, we verify that at large interatomic distances, specifically for $R_{12} = 100\,\text{Å}$ and $\eta = 0.02$, the parameter τ assumes the value $\tau \sim 1.9 > 1$. While this does not entirely rule out the use of a Taylor expansion around $\tau = 0$, it indicates that a more careful analysis is required to assess the convergence and accuracy of such an approximation. For this aim, we introduce the approximate expression of the integrand:

$$\mathscr{I}^{(l_1,l_2)}_{\text{appr}}(\bar{k};\gamma,\tau,\eta) = \mathscr{I}(\bar{k};0,0,\eta) + \sum_{l_1=1}^{m_\gamma}\sum_{l_2=1}^{m_\tau} \partial_\gamma^{l_1}\partial_\tau^{l_2}\mathscr{I}(\bar{k};\gamma,\tau,\eta)\Big|_{\gamma=0,\,\tau=0}\gamma^{l_1}\tau^{l_2}. \tag{S71}$$

As a metric for the quality of the approximation, we consider

$$\frac{\left|\delta\mathscr{I}(\bar{k};\gamma(R_{12}),\tau(R_{12}),\eta)\right|}{\left|\int_0^1 \mathscr{I}(\bar{k};\gamma(R_{12}),\tau(R_{12}),\eta)\,\mathrm{d}\bar{k}\right|}, \qquad \text{with} \qquad \delta\mathscr{I}(\bar{k};\gamma(R_{12}),\tau(R_{12}),\eta) = \mathscr{I}(\bar{k};\gamma,\tau,\eta) - \mathscr{I}^{(l_1,l_2)}_{\text{appr}}(\bar{k};\gamma,\tau,\eta). \tag{S72}$$

The maximum value of this observable over the interval $[0,1]$ for $\bar{k}$ provides an upper bound on the relative error introduced by truncating the Taylor expansion at a given order in γ and τ, in accordance with the mean value theorem for integrals. For instance, truncating the expansion at the order $m_\gamma = 3$ and $\tau = 7$ we obtain

$$\begin{aligned}
\mathscr{I}(\bar{k};\gamma(R_{12}),\tau(R_{12}),\eta) \simeq\ & \gamma\Big(-\frac{x^4\tau^2}{15\,(k_Q+x)^2} + \frac{x^6\tau^4}{210\,(k_Q+x)^2} - \frac{x^8\tau^6}{7560\,(k_Q+x)^2} + \mathscr{O}(\tau^7)\Big) \\
& + \gamma^3\Big(-\frac{x^2(5k_Q^2+4xk_Q+x^2)}{4\,(k_Q+x)^4} + \frac{x^4(5k_Q^2+4xk_Q+x^2)\tau^2}{60\,(k_Q+x)^4} - \frac{x^6(5k_Q^2+4xk_Q+x^2)\tau^4}{3360\,(k_Q+x)^4} + \mathscr{O}(\tau^7)\Big) \\
& + \gamma^5\Big(-\frac{5x^2(63k_Q^4+122xk_Q^3+102x^2k_Q^2+42x^3k_Q+7x^4)}{64\,(k_Q+x)^6} \\
& + \frac{7x^4(63k_Q^4+122xk_Q^3+102x^2k_Q^2+42x^3k_Q+7x^4)\tau^2}{960\,(k_Q+x)^6} \\
& - \frac{13x^6(63k_Q^4+122xk_Q^3+102x^2k_Q^2+42x^3k_Q+7x^4)\tau^4}{53760\,(k_Q+x)^6} + \\
& + \frac{x^8(63k_Q^4+122xk_Q^3+102x^2k_Q^2+42x^3k_Q+7x^4)\tau^6}{241920\,(k_Q+x)^6} + \mathscr{O}(\tau^7)\Big)
\end{aligned} \tag{S73}$$

We remark that

- The dependency on the mutual distances R_{12} is fully encoded in $\gamma \propto R_{12}^{-3}$ and $\tau \propto R_{12}$;
- For any given order l_γ there exists $l_\tau > 3l_\gamma$ such that positive powers of R_{12} can be generated;

- The term leading to R_{12}^{-1} dependency comes uniquely from $l_\gamma = 1$ and $l_\tau = 2$. This means that such an interaction *requires to take into account for retardation effects*.

Based on this analysis, we find that choosing $m_\gamma = 3$ and $m_\tau = 3$ yields a reasonable approximation for the values of k_M considered in the main text ($\eta \leq 2 \times 10^{-2}$). Making explicit the dependency on R_{12} of τ and γ and recasting the terms of the expansion in powers of R_{12} we obtain an approximated expression for the interaction potential

$$\mathscr{I}_{\text{appr}}^{(3,3)}(\bar{k}, \gamma(R_{12}), \tau(R_{12}), \eta) = \left(\frac{c_1^{(\mathscr{I})}(\bar{k})}{R_{12}} + \frac{c_7^{(\mathscr{I})}(\bar{k})}{R_{12}^7} + \frac{c_9^{(\mathscr{I})}(\bar{k})}{R_{12}^9} \right) \tag{S74}$$

where the coefficient reads as

$$c_1^{(\mathscr{I})}(\bar{k}) = -\frac{\mathscr{A}_0 k_M^2 \bar{k}^4}{15(\bar{k} + \bar{k}_Q)^2} \tag{S75}$$

$$c_7^{(\mathscr{I})}(\bar{k}) = \frac{\mathscr{A}_0^3 \bar{k}^4 k_M^2 \left(\bar{k}^2 + 4\bar{k}\bar{k}_Q + 5\bar{k}_Q^2 \right)}{60(\bar{k} + \bar{k}_Q)^4} \tag{S76}$$

$$c_9^{(\mathscr{I})}(\bar{k}) = -\frac{\mathscr{A}_0^3 \bar{k}^2 \left(\bar{k}^2 + 4\bar{k}\bar{k}_Q + 5\bar{k}_Q^2 \right)}{4(\bar{k} + \bar{k}_Q)^4} \tag{S77}$$

After integrating the expression in Eq.(S74) with respect to $\bar{k}$, we obtain

$$\Delta V_{12}^{\text{int}} = \Delta V \left[\frac{c_1}{R_{12}} + \frac{c_7}{R_{12}^7} + \frac{c_9}{R_{12}^9} \right] . \tag{S78}$$

where the coefficient reads

$$c_1 = \frac{\mathscr{A}_0 k_M^2 \left[2(6\bar{k}_Q^2 + 3\bar{k}_Q - 1)\bar{k}_Q + 1 - 24(\bar{k}_Q + 1)\bar{k}_Q^3 \coth^{-1}(2\bar{k}_Q + 1) \right]}{45(\bar{k}_Q + 1)} \tag{S79}$$

$$c_7 = -\frac{\mathscr{A}_0^3 k_M^2 (3\bar{k}_Q + 1)}{180(\bar{k}_Q + 1)^3} \tag{S80}$$

$$c_9 = \frac{\mathscr{A}_0^3 (3 + 5\bar{k}_Q)}{12(1 + \bar{k}_Q)^3} \tag{S81}$$

For all the choices of k_M and the QDOs parameters that reproduce the Ar atom electric response properties, $c_1 > 0$ so that the $1/R$ term is repulsive.

We notice here that the coefficient in Eq.(S79) are expressed as a function of the QDO polarizability $\mathscr{A}_0$. However, the polarizability can be expressed as a function of the fine structure constant α_{fsc} as $\mathscr{A}_0 = \alpha_{\text{fsc}} \hbar c / m\omega^2$ so that the coefficients can be rewritten as follows in different system of units:

- In SI units

$$c_1 = \alpha_{\rm fsc} \frac{\hbar c}{m\omega^2} \frac{k_M^2 \left[2(6\bar{k}_Q^2 + 3\bar{k}_Q - 1)\bar{k}_Q + 1 - 24(\bar{k}_Q + 1)\bar{k}_Q^3 \coth^{-1}(2\bar{k}_Q + 1)\right]}{45(\bar{k}_Q + 1)} \tag{S82}$$

$$c_7 = -\alpha_{\rm fsc}^3 \left(\frac{\hbar c}{m\omega^2}\right)^3 \frac{k_M^2\,(3\bar{k}_Q + 1)}{180(\bar{k}_Q + 1)^3} \tag{S83}$$

$$c_9 = \alpha_{\rm fsc}^3 \left(\frac{\hbar c}{m\omega^2}\right)^3 \frac{k_M^2\,(3\bar{k}_Q + 1)}{180(\bar{k}_Q + 1)^3} \tag{S84}$$

- In natural units

$$c_1 = \alpha_{\rm fsc} \frac{1}{m\omega^2} \frac{k_M^2 \left[2(6\bar{k}_Q^2 + 3\bar{k}_Q - 1)\bar{k}_Q + 1 - 24(\bar{k}_Q + 1)\bar{k}_Q^3 \coth^{-1}(2\bar{k}_Q + 1)\right]}{45(\bar{k}_Q + 1)} \tag{S85}$$

$$c_7 = -\alpha_{\rm fsc}^3 \left(\frac{1}{m\omega^2}\right)^3 \frac{k_M^2\,(3\bar{k}_Q + 1)}{180(\bar{k}_Q + 1)^3} \tag{S86}$$

$$c_9 = \alpha_{\rm fsc}^3 \left(\frac{1}{m\omega^2}\right)^3 \frac{k_M^2\,(3\bar{k}_Q + 1)}{180(\bar{k}_Q + 1)^3} \tag{S87}$$

- in atomic units

$$c_1 = \frac{1}{m\omega^2} \frac{k_M^2 \left[2(6\bar{k}_Q^2 + 3\bar{k}_Q - 1)\bar{k}_Q + 1 - 24(\bar{k}_Q + 1)\bar{k}_Q^3 \coth^{-1}(2\bar{k}_Q + 1)\right]}{45(\bar{k}_Q + 1)} \tag{S88}$$

$$c_7 = -\left(\frac{1}{m\omega^2}\right)^3 \frac{k_M^2\,(3\bar{k}_Q + 1)}{180(\bar{k}_Q + 1)^3} \tag{S89}$$

$$c_9 = \left(\frac{1}{m\omega^2}\right)^3 \frac{k_M^2\,(3\bar{k}_Q + 1)}{180(\bar{k}_Q + 1)^3} \tag{S90}$$

5 Energy-Shift due to interaction Hamiltonian involving A^2

The interaction term $H_{\rm int}^{(2)} = \sum_{a=1,2} \frac{q^2}{2m} A^2(\boldsymbol{R}_a)$ represents two-photon processes, in which the interaction occurs through the simultaneous exchange of two photons between two centers. Since the vector potential A is linear in terms of creation and annihilation operators of photons $(a_{k,\lambda}^\dagger, a_{k,\lambda})$, A^2 contains four types of combinations of $a_{k,\lambda}$ and $a_{k,\lambda}^\dagger$, namely $a_{k,\lambda}^\dagger a_{k,\lambda}^\dagger$, $a_{k,\lambda}^\dagger a_{k,\lambda}$, $a_{k,\lambda} a_{k,\lambda}^\dagger$, and $a_{k,\lambda} a_{k,\lambda}$. Therefore, the first order of perturbation merely contributes to self-energies that are independent of R. From the second-order perturbation, we obtain the leading-order energy shift due to

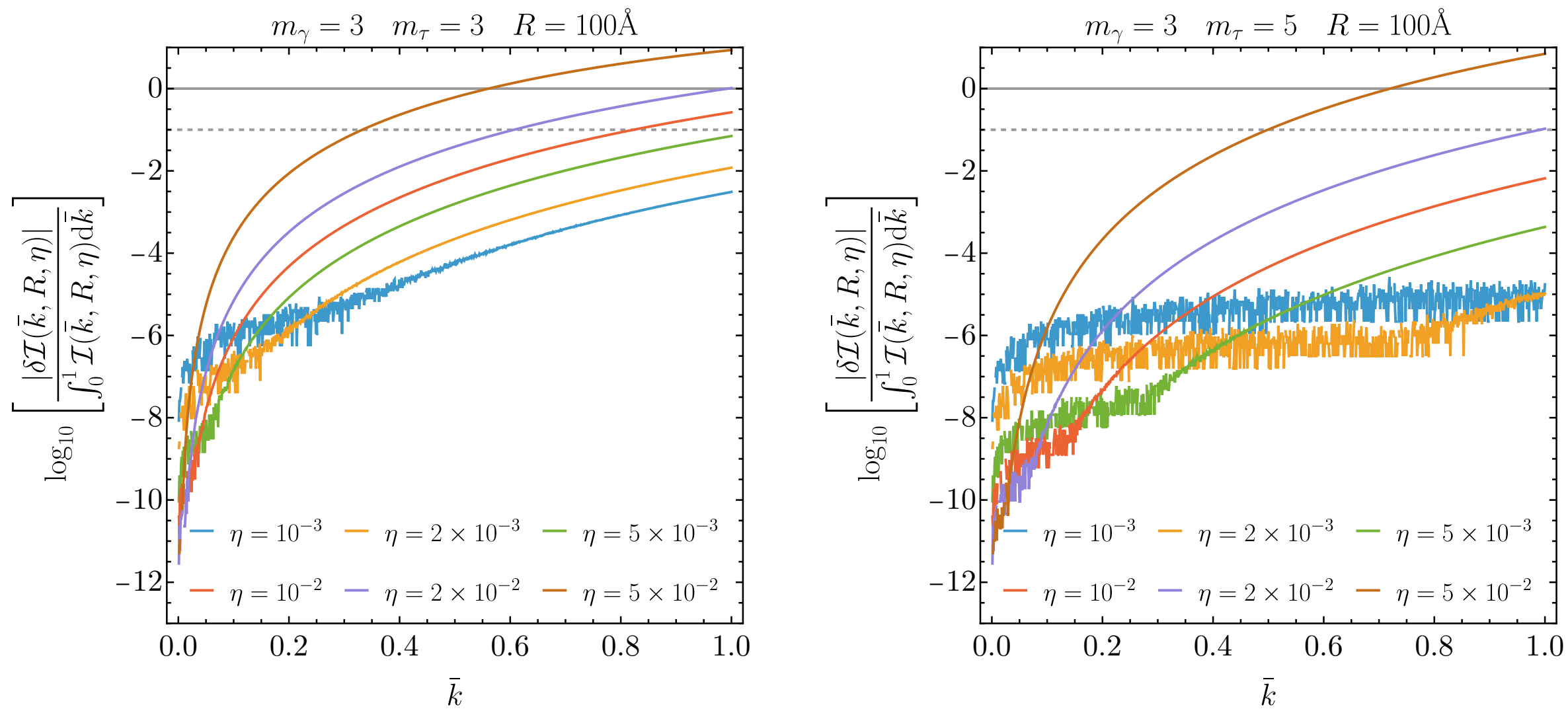

Figure S1: **Error analysis in the approximation of** $\mathscr{I}_{\text{appr}}(\bar{k};\gamma,\tau,\eta)$.

the matter-field interaction Hamiltonian $H_{\text{int}}^{(2)}$ as follows:

$$\Delta E_2 = \sum_{I\neq 0} \frac{\langle 0|H_{int}^{(2)}|I\rangle\langle I|H_{int}^{(2)}|0\rangle}{E_0 - E_I} \,, \tag{S91}$$

where in the intermediate state $|I\rangle$, the field has to be in a two-photon excited state $|1_{k,\lambda}\, 1_{k',\lambda'}\rangle$, and the molecular state (MBD state) has to be in the ground state in order to result in non-vanishing energy shifts, hence, $E_0 - E_I = -\hbar c(k+k')$. Therefore we have

$$\begin{aligned}\langle I|H_{int}^{(2)}|0\rangle &= \langle I| \sum_{a=1,2}\sum_{k,\lambda}\sum_{k',\lambda'} \frac{q_a^2}{2m_a}\frac{\hbar}{2\varepsilon_0 cV}\frac{1}{\sqrt{kk'}}[\boldsymbol{e}(k,\lambda)\cdot\boldsymbol{e}(k',\lambda')]a_{k,\lambda}^\dagger a_{k',\lambda'}^\dagger e^{-i(\boldsymbol{k}+\boldsymbol{k}')\cdot\boldsymbol{R}_a}\,|0\rangle \\ &= \sum_{a=1,2}\sum_{k,\lambda}\sum_{k',\lambda'} \frac{q_a^2}{2m_a}\frac{\hbar}{2\varepsilon_0 cV}\frac{1}{\sqrt{kk'}}[\sum_{i=x,y,z} e_i(k,\lambda)e_i(k',\lambda')]e^{-i(\boldsymbol{k}+\boldsymbol{k}')\cdot\boldsymbol{R}_a}\,,\end{aligned} \tag{S92}$$

thus

$$\begin{aligned}\Delta E_2 = \sum_{I\neq 0}\frac{\langle 0|H_{int}^{(2)}|I\rangle\langle I|H_{int}^{(2)}|0\rangle}{E_0-E_I} &= -\sum_{a,b=1}^{2}\sum_{i,j=x,y,z}\sum_{k,k'}\frac{q_a^2 q_b^2}{4m_a m_b}\left(\frac{\hbar}{2\varepsilon_0 cV}\right)^2\frac{1}{\hbar c\, kk'(k+k')}e^{i(\boldsymbol{k}+\boldsymbol{k}')\cdot(\boldsymbol{R}_b-\boldsymbol{R}_a)} \\ &\quad\times[\sum_{\lambda=1}^{2} e_i(k,\lambda)e_j(k,\lambda)]\,[\sum_{\lambda'=1}^{2} e_i(k',\lambda')e_j(k',\lambda')] \\ &= -\sum_{a,b=1}^{2}\sum_{i,j=x,y,z}\sum_{k,k'}\frac{q_a^2 q_b^2}{4m_a m_b}\left(\frac{\hbar}{2\varepsilon_0 cV}\right)^2\frac{1}{\hbar c\, kk'(k+k')}e^{i(\boldsymbol{k}+\boldsymbol{k}')\cdot(\boldsymbol{R}_b-\boldsymbol{R}_a)}[\delta_{ij}-\hat{k}_i\hat{k}_j]\,[\delta_{ij}-\hat{k}'_i\hat{k}'_j]\,,\end{aligned} \tag{S93}$$

where in the last line of (S93) the expressions inside the square brackets result from summing over polarizations of the field. In the continuum limit of the quantization volume V, the sums over the wavenumbers k and k' are replaced by integrals, *i.e* $\frac{1}{V}\sum_k \to \int \frac{d^3k}{(2\pi)^3}$. Using the spherical coordinates for the integrals over the wavevectors $\boldsymbol{k}$ and $\boldsymbol{k}'$, we arrive at

$$\Delta E_2 = -\sum_{a,b=1}^{2}\sum_{i,j=x,y,z}\frac{q_a^2 q_b^2}{4m_a m_b}\frac{\hbar}{4\varepsilon_0^2 c^3}\left\{\frac{1}{8\pi^3}\int_0^\infty k\,dk\int e^{i\boldsymbol{k}\cdot(\boldsymbol{R}_b-\boldsymbol{R}_a)}[\delta_{ij}-\hat{k}_i\hat{k}_j]d\Omega\right.$$
$$\left.\left\{\frac{1}{8\pi^3}\int_0^\infty \frac{k'}{(k+k')}dk'\int e^{i\boldsymbol{k}'\cdot(\boldsymbol{R}_b-\boldsymbol{R}_a)}[\delta_{ij}-\hat{k}'_i\hat{k}'_j]d\Omega'\right\}\right\}\ . \tag{S94}$$

When $a=b$, the virtual transitions of the total system in the two-step process of the second-order perturbation correspond to the creation and annihilation of a pair of virtual photons at the same center, hence giving an energy shift that is independent of the molecular state and can be considered as a constant shift to all energy levels. When $a\neq b$, there are two possibilities: $(a,b)=(1,2)$ that indicates creation of two virtual photons at $\boldsymbol{R}_1$ and annihilation of them at $\boldsymbol{R}_2$, or $(a,b)=(2,1)$ for the opposite creation/annihilation process. The energy shifts resulting from such processes depend on the distance between the photon-creation/annihilation centers, hence depend on the molecular state. Therefore, for $a\neq b$ we have $\boldsymbol{R}_b-\boldsymbol{R}_a=\pm\boldsymbol{R}$ where $\boldsymbol{R}=\boldsymbol{R}_2-\boldsymbol{R}_1$, and we end up with the known angular integral

$$\tau_{ij}(kR)=\frac{1}{4\pi}\int e^{\pm i\boldsymbol{k}\cdot\boldsymbol{R}}[\delta_{ij}-\hat{k}_i\hat{k}_j]d\Omega=(\delta_{ij}-R_iR_j)\frac{\sin(kR)}{kR}+(\delta_{ij}-3R_iR_j)\left[\frac{\cos(kR)}{k^2R^2}-\frac{\sin(kR)}{k^3R^3}\right]\ . \tag{S95}$$

Using this integral identity expression (S94) reduces to

$$\Delta E_2=-2\sum_{i,j=x,y,z}\frac{q_1^2q_2^2}{4m_1m_2}\frac{\hbar}{16\pi^4\varepsilon_0^2c^3}\left\{\int_0^\infty k\,\tau_{ij}(kR)\,dk\left\{\int_0^\infty\frac{k'}{(k+k')}\tau_{ij}(k'R)\,dk'\right\}\right\}\ . \tag{S96}$$

To decouple the integrals, we use the integral identity

$$\frac{1}{k+k'}=\int_0^\infty e^{-u(k+k')}\,du\ , \tag{S97}$$

hence equation (S96) becomes

$$\Delta E_2=-2\sum_{i,j=x,y,z}\frac{q_a^2q_b^2}{4m_am_b}\frac{\hbar}{16\pi^4\varepsilon_0^2c^3}\int_0^\infty\left\{\left[\int_0^\infty k\,\tau_{ij}(kR)\,e^{-uk}\,dk\right]\left[\int_0^\infty k'\,\tau_{ij}(k'R)\,e^{-uk'}\,dk'\right]\right\}du$$
$$=-\frac{q_a^2q_b^2}{2m_am_b}\frac{\hbar}{16\pi^4\varepsilon_0^2c^3}\sum_{i,j=x,y,z}\int_0^\infty\left[F_{ij}(u,R)\right]^2du\ , \tag{S98}$$

where

$$
\begin{aligned}
F_{ij}(u,R) &= \int_0^\infty k\,\tau_{ij}(kR)\,e^{-uk}\,dk \\
&= \int_0^\infty e^{-uk}\left\{(\delta_{ij}-R_iR_j)\frac{\sin(kR)}{R} + (\delta_{ij}-3R_iR_j)\left[\frac{\cos(kR)}{kR^2} - \frac{\sin(kR)}{k^2R^3}\right]\right\} dk \\
&= \frac{(\delta_{ij}-R_iR_j)}{u^2+R^2} + \frac{(\delta_{ij}-3R_iR_j)}{R}\int_0^\infty e^{-uk}\left[\frac{\cos(kR)}{kR} - \frac{\sin(kR)}{k^2R^2}\right] dk \,. \qquad \text{(S99)}
\end{aligned}
$$

Taking the remaining integral in the definition of $F_{ij}(u,R)$ is challenging. We notice that the integrand in the last line of equation (S99) is, apart from the exponential part, a first-order derivative with respect to k, *i.e.*

$$
\left[\frac{\cos(kR)}{kR} - \frac{\sin(kR)}{k^2R^2}\right] = \frac{1}{R}\frac{d}{dk}\left(\frac{\sin(kR)}{kR}\right) \,. \qquad \text{(S100)}
$$

Using the above equality, we calculate the integral as follows

$$
\begin{aligned}
&\frac{(\delta_{ij}-3R_iR_j)}{R}\int_0^\infty e^{-uk}\left[\frac{\cos(kR)}{kR} - \frac{\sin(kR)}{k^2R^2}\right] dk = \frac{(\delta_{ij}-3R_iR_j)}{R^2}\int_0^\infty e^{-uk}\left[\frac{d}{dk}\left(\frac{\sin(kR)}{kR}\right)\right] dk \\
=&\frac{(\delta_{ij}-3R_iR_j)}{R^2}\left[e^{-uk}\frac{\sin(kR)}{kR}\right]_0^\infty + \frac{(\delta_{ij}-3R_iR_j)}{R^2}\int_0^\infty \frac{\sin(kR)}{kR}ue^{-uk}\,dk \\
=&(\delta_{ij}-3R_iR_j)\left[-\frac{1}{R^2} + \frac{u}{R^3}\arctan(\frac{R}{u})\right] \,. \qquad \text{(S101)}
\end{aligned}
$$

Therefore, $F_{ij}(u,R)$ is obtained as

$$
F_{ij}(u,R) = \frac{(\delta_{ij}-R_iR_j)}{u^2+R^2} + (\delta_{ij}-3R_iR_j)\left[-\frac{1}{R^2} + \frac{u}{R^3}\arctan(\frac{R}{u})\right] \,. \qquad \text{(S102)}
$$

As before, we assume the two QDOs are placed on the z-axis such that $\boldsymbol{R}_2 - \boldsymbol{R}_1 = \boldsymbol{R} = R\hat{\boldsymbol{z}}$. With this assumption, we perform the sums over i and j to obtain

$$
\begin{aligned}
\sum_{i,j=x,y,z}\left[F_{ij}(u,R)\right]^2 = &\frac{6}{R^4} - \frac{4}{R^2\,(R^2+u^2)} + \frac{2}{(R^2+u^2)^2} - \frac{12u}{R^5}\arctan\left(\frac{R}{u}\right) + \frac{4u}{R^3\,(R^2+u^2)}\arctan\left(\frac{R}{u}\right) \\
&+ \frac{6u^2}{R^6}\arctan\left(\frac{R}{u}\right)^2 \,. \qquad \text{(S103)}
\end{aligned}
$$

Replacing the sum (S103) into (S98) and taking the u-integral we arrive at the interaction energy

$$
\Delta E_2 = -\frac{q_a^2 q_b^2}{2m_a m_b}\frac{\hbar}{16\pi^4\varepsilon_0^2 c^3}\frac{\pi}{2R^3} \,. \qquad \text{(S104)}
$$

REFERENCES

1. H. B. Casimir, On the attraction between two perfectly conducting plates. *Proc. Kon. Ned. Akad. Wet.* **51**, 793 (1948).

2. P. W. Milonni, *The Quantum Vacuum: An Introduction to Quantum Electrodynamics* (Academic Press, 2013).

3. D.P. Craig, T. Thirunamachandran, *Molecular Quantum Electrodynamics: An Introduction to Radiation-Molecule Interactions* (Courier Corporation, 1998).

4. A. Tkatchenko, Current understanding of van der Waals effects in realistic materials. *Adv. Funct. Mater.* **25**, 2054–2061 (2015).

5. A. Salam, *Molecular Quantum Electrodynamics: Long-Range Intermolecular Interactions* (John Wiley & Sons, 2009).

6. A. Salam, *Non-Relativistic QED Theory of the van der Waals Dispersion Interaction* (Springer, 2016).

7. S. Y. Buhmann, *Dispersion Forces I: Macroscopic quantum electrodynamics and ground-state Casimir, Casimir–Polder and van der Waals forces*, vol. 247 (Springer, 2013).

8. R. Passante, Dispersion interactions between neutral atoms and the quantum electrodynamical vacuum. *Symmetry* **10**, 735 (2018).

9. M. Gori, P. Kurian, A. Tkatchenko, Second quantization of many-body dispersion interactions for chemical and biological systems. *Nat. Commun.* **14**, 8218 (2023).

10. M. R. Karimpour, D. V. Fedorov, A. Tkatchenko, Quantum framework for describing retarded and nonretarded molecular interactions in external electric fields. *Phys. Rev. Res.* **4**, 013011 (2022).

11. M. R. Karimpour, D. V. Fedorov, A. Tkatchenko, Molecular interactions induced by a static electric field in quantum mechanics and quantum electrodynamics. *J. Phys. Chem. Lett.* **13**, 2197–2204 (2022).

12. J. Flick, M. Ruggenthaler, H. Appel, A. Rubio, Atoms and molecules in cavities, from weak to strong coupling in quantum-electrodynamics (QED) chemistry. *Proc. Natl. Acad. Sci. U.S.A* **114**, 3026–3034 (2017).

13. R. R. Riso, T. S. Haugland, E. Ronca, H. Koch, Molecular orbital theory in cavity QED environments. *Nat. Commun.* **13**, 1368 (2022).

14. P. Forn-Díaz, L. Lamata, E. Rico, J. Kono, E. Solano, Ultrastrong coupling regimes of light-matter interaction. *Rev. Mod. Phys.* **91**, 025005 (2019).

15. Y. Ashida, A. İmamoğlu, E. Demler, Cavity quantum electrodynamics at arbitrary light-matter coupling strengths. *Phys. Rev. Lett.* **126**, 153603 (2021).

16. B. Xiang, W. Xiong, Molecular polaritons for chemistry, photonics and quantum technologies. *Chem. Rev.* **124**, 2512–2552 (2024).

17. T. S. Haugland, E. Ronca, E. F. Kjønstad, A. Rubio, H. Koch, Coupled cluster theory for molecular polaritons: Changing ground and excited states. *Phys. Rev. X* **10**, 041043 (2020).

18. M. Hertzog, M. Wang, J. Mony, K. Börjesson, Strong light-matter interactions: A new direction within chemistry. *Chem. Soc. Rev.* **48**, 937–961 (2019).

19. A. Mandal, M. A. D. Taylor, B. M. Weight, E. R. Koessler, X. Li, P. Huo, Theoretical advances in polariton chemistry and molecular cavity quantum electrodynamics. *Chem. Rev.* **123**, 9786–9879 (2023).

20. A. Tkatchenko, R. A. DiStasio Jr., R. Car, M. Scheffler, Accurate and efficient method for many-body van der Waals interactions. *Phys. Rev. Lett.* **108**, 236402 (2012).

21. R. A. DiStasio Jr., V. V. Gobre, A. Tkatchenko, Many-body van der Waals interactions in molecules and condensed matter. *J. Phys. Condens. Matter* **26**, 213202 (2014).

22. L. M. Woods, D. A. R. Dalvit, A. Tkatchenko, P. Rodriguez-Lopez, A. W. Rodriguez, R. Podgornik, Materials perspective on Casimir and van der Waals interactions. *Rev. Mod. Phys.* **88**, 045003 (2016).

23. J. Hermann, R. A. DiStasio Jr., A. Tkatchenko, First-principles models for van der Waals interactions in molecules and materials: Concepts, theory, and applications. *Chem. Rev.* **117**, 4714–4758 (2017).

24. H. B. G. Casimir, D. Polder, The influence of retardation on the London-van der Waals forces. *Phys. Rev.* **73**, 360–372 (1948).

25. M. D. Crisp, Interaction of a charged harmonic oscillator with a single quantized electromagnetic field mode. *Phys. Rev. A* **44**, 563–573 (1991).

26. M. J. Renne, Retarded van der Waals interaction in a system of harmonic oscillators. *Physica* **53**, 193–209 (1971).

27. G. Compagno, R. Passante, F. Persico, *Atom-Field Interactions and Dressed Atoms* (Cambridge Univ. Press, 2009).

28. F. Ciccarello, E. Karpov, R. Passante, Exactly solvable model of two three-dimensional harmonic oscillators interacting with the quantum electromagnetic field: The far-zone Casimir-Polder potential. *Phys. Rev. A* **72**, 052106 (2005).

29. M. E. Peskin, D. V. Schroeder, *An Introduction to Quantum Field Theory* (Addison-Wesley, 2018).

30. M. K. Svendsen, M. Ruggenthaler, H. Hübener, C. Schäfer, M. Eckstein, A. Rubio, S. Latini, Effective equilibrium theory of quantum light-matter interaction in cavities for extended systems and the long wavelength approximation. *Commun. Phys.* **8**, 425 (2025).

31. A. Tkatchenko, A. Ambrosetti, R. DiStasio, Interatomic methods for the dispersion energy derived from the adiabatic connection fluctuation-dissipation theorem. *J. Chem. Phys.* **138**, 074106 (2013).

32. J. Preto, M. Pettini, J. A. Tuszynski, Possible role of electrodynamic interactions in long-distance biomolecular recognition. *Phys. Rev. E* **91**, 052710 (2015).

33. L. Gomberoff, R. McLone, E. A. Power, Long-range retarded potentials between molecules. *J. Chem. Phys.* **44**, 4148–4153 (1966).

34. P. Barcellona, R. Passante, L. Rizzuto, S. Y. Buhmann, van der Waals interactions between excited atoms in generic environments. *Phys. Rev. A* **94**, 012705 (2016).

35. A. Szabo, N. S. Ostlund, *Modern Quantum Chemistry: Introduction to Advanced Electronic Structure Theory* (Courier Corporation, 1996).

36. P. Szabó, S. Góger, J. Charry, M. R. Karimpour, D. V. Fedorov, A. Tkatchenko, Four-dimensional scaling of dipole polarizability in quantum systems. *Phys. Rev. Lett.* **128**, 070602 (2022).

37. S. Góger, M. R. Karimpour, A. Tkatchenko, Four-dimensional scaling of dipole polarizability: From single-particle models to atoms and molecules. *J. Chem. Theory Comput.* **20**, 6621–6631 (2024).

38. A. Ambrosetti, N. Ferri, R. A. DiStasio Jr., A. Tkatchenko, Wavelike charge density fluctuations and van der Waals interactions at the nanoscale. *Science* **351**, 1171–1176 (2016).

39. A. A. Geraci, S. B. Papp, J. Kitching, Short-range force detection using optically cooled levitated microspheres. *Phys. Rev. Lett.* **105**, 101101 (2010).

40. A. O. Sushkov, W. J. Kim, D. A. R. Dalvit, S. K. Lamoreaux, New experimental limits on non-Newtonian forces in the micrometer range. *Phys. Rev. Lett.* **107**, 171101 (2011).

41. J. Manley, C. A. Condos, S. Schlamminger, J. R. Pratt, D. J. Wilson, W. A. Terrano, Microscale torsion resonators for short-range gravity experiments. *Phys. Rev. D* **110**, 122005 (2024).

42. L. Berezhiani, J. Khoury, Emergent long-range interactions in Bose-Einstein condensates. *Phys. Rev. D* **99**, 076003 (2019).

43. D. O'dell, S. Giovanazzi, G. Kurizki, V. M. Akulin, Bose-Einstein condensates with $1/r$ interatomic attraction: Electromagnetically induced "gravity". *Phys. Rev. Lett.* **84**, 5687 (2000).

44. A. P. Jones, J. Crain, V. P. Sokhan, T. W. Whitfield, G. J. Martyna, Quantum Drude oscillator model of atoms and molecules: Many-body polarization and dispersion interactions for atomistic simulation. *Phys. Rev. B* **87**, 144103 (2013).
